# Early urine proteome changes in the Walker-256 tail-vein injection rat model

**DOI:** 10.1038/s41598-019-50301-1

**Published:** 2019-09-24

**Authors:** Jing Wei, Na Ni, Wenshu Meng, Youhe Gao

**Affiliations:** 1 Department of Biochemistry and Molecular Biology, Beijing Normal University, Gene Engineering Drug and Biotechnology Beijing Key Laboratory, Beijing, 100875 China; 2 Department of Biochemistry and Molecular Biology, College of Basic Medicine, Chongqing Medical University, Chongqing, 400016 China

**Keywords:** Lung cancer, Diagnostic markers

## Abstract

Detection of cancer at its early stage is important for treatment. Urine, which is not regulated by homeostatic mechanisms, reflects early systemic changes throughout the whole body and can be used for the early detection of cancer. In this study, the Walker-256 tail-vein injection rat model was established to find whether the urine proteome could reflect early changes if tumor grown in lung. Urine samples from the control group (n = 7) and Walker-256 tail-vein injection group (n = 7) on days 2, 4, 6 and 9 were analyzed by label-free proteomic quantitative methods. On day 2, when lung tumor nodules did not appear, 62 differential proteins were identified. They were associated with epithelial cell differentiation, regulation of immune system processes and the classical complement activation pathway. On day 4, when lung tumor nodules appeared, 72 differential proteins were identified. They were associated with the innate immune response and positive regulation of phagocytosis. On day 6, when body weight began to decrease, 117 differential proteins were identified. On day 9, the identified 125 differential proteins were associated with the B cell receptor signaling pathway and the positive regulation of B cell activation. Our results indicate that (1) the urine proteome changed even on the second day after tail-vein injection of Walker-256 cells and that (2) compared to previous studies, the urine proteomes were different when the same cancer cells were grown in different organs.

## Introduction

Cancer metastasis is a process in which cancer cells are disseminated from the primary tumor tissue to different sites through blood vessels and lymphatic vessels. The lung, brain, bone and liver are common metastatic organs in cancer patients^1^. Distant organ metastasis accounts for most cancer morbidity and mortality and approximately 90% of cancer deaths^2^ are usually accompanied by a poor 5-year survival rate as well as limited treatment strategies^3^. Due to special lung-specific immunoregulatory mechanisms, tumor colonization occurs more readily in an immunologically permissive environment^4^. Therefore, many cancer metastases such as those from breast cancer and malignant melanoma occur more easily in the lung. The early detection of cancer metastasis is still elusive, as finding and predicting specific distant metastatic organs is difficult, especially in early-stage cancer without clinical symptoms. Therefore, early detection of cancer metastasis can significantly improve the survival rate and effective therapies for cancer patients and helps in monitoring cancer metastasis progression over time.

Biomarkers are measurable changes associated with the physiological or pathophysiological processes of disease and usually derive from tissue, blood and tumor cells^5^. Because of the homeostatic mechanisms in the internal environment, the levels of important factors in the blood tend to be stable to protect the stability of the internal environment^6^. Without the control of homeostatic mechanisms, urine can accumulate systemic changes from the whole body, which has the potential to reflect the small pathophysiological changes from disease^7^. Therefore, urine has the potential to reflect early changes in disease. However, in clinical patients, urinary proteins are easily affected by some complicated factors, such as gender, age, and medications. Therefore, using animal models can help determine the direct relationship between urine protein changes and related diseases such as cancer metastasis, because the genetic and environmental factors are minimized^8^.

Various studies have applied urinary proteomics to discover cancer biomarkers for the early diagnosis and monitoring of cancer^9–11^. However, most of these studies used clinical urine samples from cancer patients who had distant metastases to viscera or bone^9^. It is difficult to clinically collect the exact early stages of different cancer lung metastasis samples. Using animal models allows for the control of the exact starting point of cancer lung metastasis, which will help to identify the early candidate biomarkers in cancer lung metastasis.

**Figure 1:**
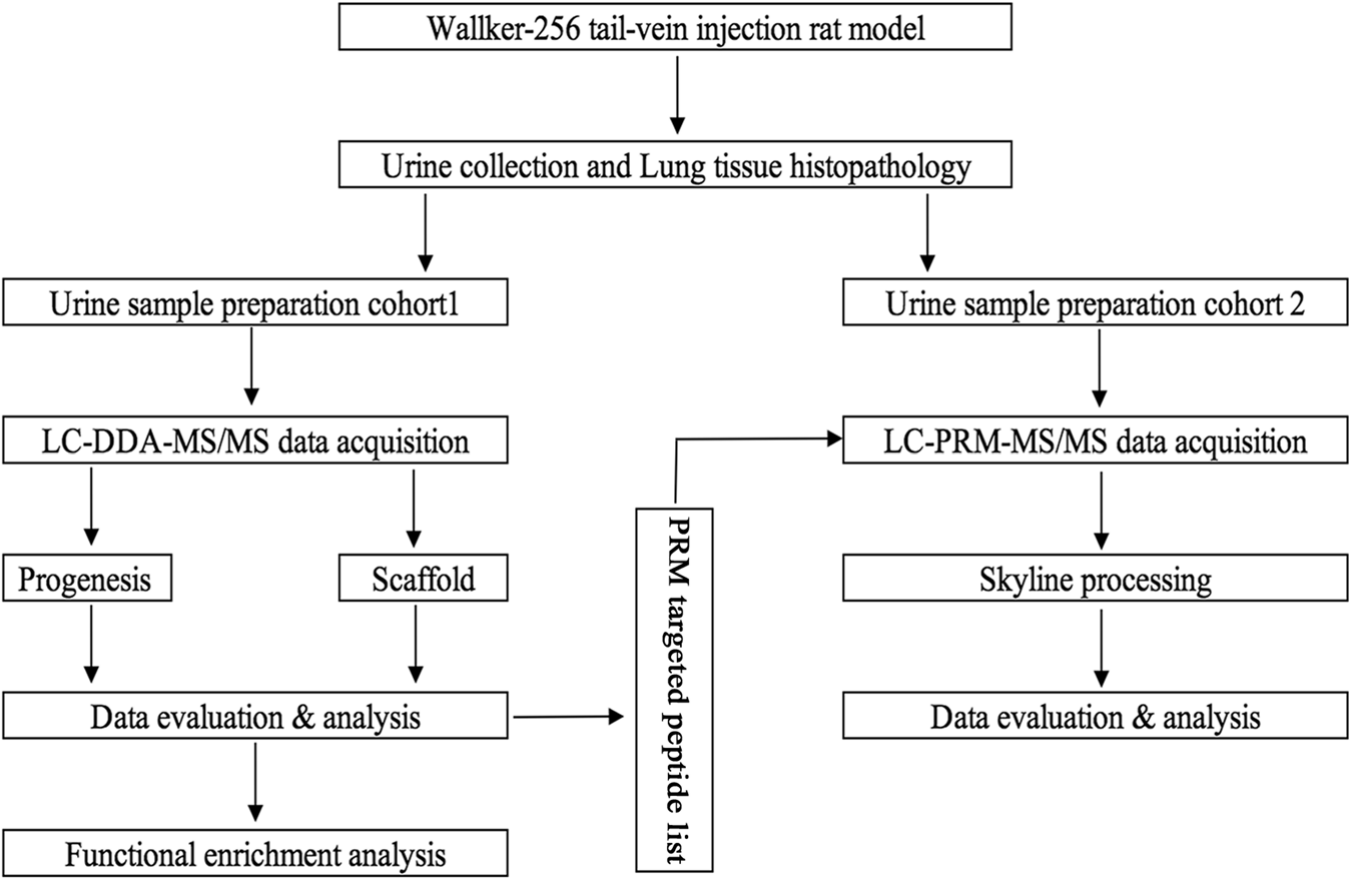
Workflow of urinary candidate biomarker discovery and validation. After tail-vein injection of Walker-256 cells, urine samples were collected on days 2, 4, 6, and 9. After urinary protein extraction and tryptic digestion, the urinary proteome was analyzed using liquid chromatography coupled with tandem mass spectrometry (LC-MS/MS) identification. The common continuous changed proteins identified on day 2 and day 4 were validated by parallel reaction monitoring (PRM)-targeted proteomics quantitative analysis.

Walker-256 cells are mammary gland carcinoma cells^12^, and the Walker-256 tail-vein injection rat model is a well-known cancer lung metastasis rat model for studies of lung metastasis progression, such as evaluating the effects of some drugs on the development of Walker-256 lung metastases^13^. In this study, the Walker-256 tail-vein injection rat model was established by injecting Walker-256 tumor cells. Urine samples were collected from the Walker-256 tail-vein injection group (n = 7) on day 2, 4, 6, 9 and the control group (n = 7) by two different mass spectrometers for candidate biomarker discovery. Then, these candidate proteins were validated by parallel reaction monitoring (PRM)-targeted quantitation analysis. This study was designed to find a panel of candidate urinary biomarkers related to the early phase of tumor cells grown in the lung. The workflow of this research is presented in Fig. 1.

## Materials and Methods

### Ethics statement

Male Wistar rats (150 ± 20 g) and Sprague-Dawley (SD) rats (70 ± 20 g) were purchased from the Beijing Vital River Laboratory Animal Technology Co, Ltd. All animals were housed with free access to a standard laboratory diet and water with indoor temperature (22 ± 1 °C) and humidity (65~70%). All animal protocols governing the experiments were approved by the Institutional Animal Care and Use Committee of Peking Union Medical College (Approved ID: ACUC-A02-2014-008). All methods in this research were performed in accordance with the guidelines and regulations.

### Rat model establishment

The Walker-256 tail-vein injection rat model was established as reported previously^13^. The Walker-256 carcinosarcoma cells were purchased from the Cell Culture Center of the Chinese Academy of Medical Sciences (Beijing, China). Briefly, male SD rats were used for ascitic tumor cell cultivation. After two cell passages, the Walker-256 tumor cells were collected, centrifuged, and resuspended in phosphate-buffered saline (PBS) for the following establishment of rat models. The cell viability was assessed by the trypan blue exclusion test^11^. Walker-256 cells were stained with 0.4% trypan blue solution and then counted using a hemocytometer. The viability was approximately 95% before the tail-vein injection.

Male Wistar rats were randomly divided into two groups: the Walker-256 tail-vein injection group (n = 24) and the control group (n = 12). The Walker-256 tail-vein injection group was injected with 2 × 10^6^ viable Walker-256 cells in 100 *μ*L of PBS. The corresponding control group was tail-vein injected with the same volume of PBS. The animals were anesthetized with sodium pentobarbital solution at 4 mg/kg before the tail-vein injection.

### Lung histopathology

For histopathology, three rats in the tail-vein injection group and three rats in the control group were randomly sacrificed on days 2, 4, 6, and 9 using an overdose of sodium pentobarbital anesthetic. Whole lung tissue was fixed in 4% formalin fixative and embedded in paraffin, and the paraffin sections (4-μm thick) were evaluated with hematoxylin and eosin (HE) staining to reveal the metastatic nodules.

### Urine collection and sample preparation

Urine samples were collected from the tail-vein injection group on days 2, 4, 6, 9 and the corresponding control group. Each rat was placed in metabolic cages overnight naturally for 10 h to collect urine without food and water. The urine samples were stored at –80 °C immediately after collection.

Urine samples were centrifuged at 12,000 g for 30 min at 4 °C to remove cell debris. Then, the supernatants were precipitated with three volumes of ethanol at −20 °C overnight. After centrifugation at 12,000 g for 30 min, the pellets were resuspended in lysis buffer (8 mol/L urea, 2 mol/L thiourea, 50 mmol/L Tris, and 25 mmol/L DTT) at 4 °C for 2 h^11^. Finally, after centrifugation at 4 °C and 12,000 g for 30 min, the supernatants of each sample were measured using the Bradford assay. The protein samples were stored at −80 °C for later use.

Urine proteins were digested with trypsin (Trypsin Gold, Mass Spec Grade, Promega, Fitchburg, Wisconsin, USA) using FASP methods^14^. Briefly, 100 *μ*g of protein was loaded onto 10-kD cutoff filter devices (Pall, Port Washington, NY) and washed with UA (8 M urea in 0.1 M Tris-HCl, pH 8.5) at 14,000 g for 40 min at 18 °C twice. Then, 25 mmol/L NH_4_HCO_3_ was added to wash the protein. Each urinary protein was subsequently denatured with 20 mM DTT at 37 °C for 1 h and then alkylated with 50 mM iodoacetamide (IAA) for 40 min in the dark. After being washed twice with UA and three times with 25 mmol/L NH_4_HCO_3_, the denatured proteins were resuspended with 25 mmol/L NH_4_HCO_3_ and digested with trypsin (enzyme to protein ratio of 1:50) at 37 °C for 14 h. Finally, these peptides were desalted using Oasis HLB cartridges (Waters, Milford, MA) and then dried by SpeedVac (Thermo Fisher Scientific, Bremen, Germany).

### LC-MS/MS analysis

Sixteen urine samples from four randomly selected Walker-256 tail-vein injection rats at four time points (day 2, 4, 6 and 9) and 4 urine samples from the corresponding control group were chosen for MS analysis. Digested peptides were redissolved in 0.1% formic acid and separated on a reverse-phase C18 self-packed capillary LC column (75 μm × 100 mm, 3 μm) using a Waters ultra-performance liquid chromatography (UPLC) system. The elution was performed in 60 min with a gradient of 5%–28% buffer B (0.1% formic acid and 99.9% acetonitrile (ACN); flow rate, 0.3 μL/min). The peptides were analyzed using an AB SCIEX (Framingham, MA, US) Triple TOF 5600 mass spectrometry (MS) system^15^. The MS data were acquired in high-sensitivity mode using the following parameters: 30 data-dependent MS/MS scans per full scan, full scans acquired at a resolution of 40,000 and MS/MS scans at a resolution of 20,000, rolling collision energy, charge-state screening (+2 to +5)^16^, dynamic exclusion (exclusion duration 30 s), an MS/MS scan range of 250–1800 m/z, and a scan time of 50 ms. Each sample was analyzed twice (2 μg each analysis).

Another twelve urine samples from three randomly selected Walker-256 tail-vein injection rats at four time points (day 2, 4, 6 and 9) and three urine samples from the corresponding control group were chosen for MS analysis. Digested peptides were redissolved in 0.1% formic acid to a concentration of 0.5 *μ*g/*μ*L. For analysis, 1 *μ*g of each peptide from an individual sample was loaded onto a trap column and separated on a reverse-phase C18 column (50 *μ*m × 150 mm, 2 *μ*m) using the EASY-nLC 1200 HPLC system (Thermo Fisher Scientific, Waltham, MA)^11^. The elution for the analytical column lasted 120 min with a gradient of 5%–28% buffer B (0.1% formic acid in 80% acetonitrile; flow rate 0.3 μl/min). Peptides were analyzed with an Orbitrap Fusion Lumos Tribrid mass spectrometer (Thermo Fisher Scientific, Waltham, MA). MS data were acquired in high-sensitivity mode using the following parameters: data-dependent MS/MS scans per full scan with top-speed mode (3 s), MS scans at a resolution of 120,000 and MS/MS scans at a resolution of 30,000 in the Orbitrap, 30% HCD collision energy, charge-state screening (+2 to +7), dynamic exclusion (exclusion duration 30 s), and a maximum injection time of 45 ms^11^. Each sample was analyzed twice.

### Database searching and label-free quantitation

All MS/MS data were searched using Mascot software (version 2.4.1, Matrix Science, London, UK) against the SwissProt rat database (released in February 2017, containing 7,992 sequences). For Triple TOF 5600, the parameters were set as follows: the fragment ion mass tolerance was set to 0.05 Da, and the parent ion tolerance was set to 0.05 Da^15^. For the Orbitrap Fusion Lumos, the parent ion tolerance was set to 10 ppm, and the fragment ion mass tolerance was set to 0.02 Da. Carbamidomethylation of cysteine was set as a fixed modification, and the oxidation of methionine was considered a variable modification. The specificity of trypsin digestion was set for cleavage after K or R, and two missed trypsin cleavage sites were allowed^11^.

Proteins from four Walker-256 tail-vein injection rats and four corresponding control group were then filtered using Progenesis LC-MS/MS software (version 4.1, Nonlinear, Newcastle upon Tyne, UK)^17^. All peptides (with Mascot score > 30 and P < 0.01) of an identified protein were included in the following quantitation. The acquired data from the MS scans were transformed and stored in peak lists using a proprietary algorithm. Features with only one charge or more than five charges were excluded from the analyses. Protein abundance was calculated from the sum of all unique peptide ion abundances for a specific protein in each run^15^, which normalized by the median abundance of the common identified proteins. The normalization of abundances was required to allow comparisons across different sample runs by this software. Proteins identified by more than one peptide were retained^15^.

Proteins from three Walker-256 tail-vein injection rats and three corresponding control group were filtered using Scaffold (version 4.7.5, Proteome Software Inc., Portland, OR). Both the peptide and protein identifications were accepted at a false discovery rate (FDR) of less than 1.0%. Proteins with at least two unique peptides were retained. Spectral counting was normalized by total spectra, and used to compare protein abundances at different time points according to the previously described procedures^18,19^.

### Statistical analysis

Average normalized abundance or spectral counts of each sample were used for statistical analysis. Proteins identified in the tail-vein injection group on days 2, 4, 6 and 9 were compared with those of the control group. Differential proteins were selected with the following criteria: fold change ≥1.5 or ≤0.67; confidence score ≥200; *P* < 0.05 by two-sided, unpaired t-test; protein spectral counts or the normalized abundance from every rat in the high-abundance group were greater than those in the low-abundance group, and the average spectral count in the high-abundance group ≥4^11^. Group differences resulting in *P* < 0.05 were considered statistically significant. All results are expressed as the mean ± standard deviation.

### Parallel reaction monitoring (PRM) mass spectrometry and data analysis

Sixteen urine samples from another four randomly selected Walker-256 tail-vein injection rats at four time points (day 2, 4, 6 and 9) and 4 urine samples from the corresponding control group were chosen for PRM analysis. Initial PRM runs were performed by DDA to define peptide retention times. PRM samples were analyzed by the EASY-nLC 1200 HPLC system (Thermo Fisher Scientific, Waltham, MA) coupled to the Orbitrap Fusion Lumos Tribrid mass spectrometer (Thermo Fisher Scientific, Waltham, MA). Peptides were pooled (2 *μ*g of each sample) for LC-MS/MS analysis to build a spectrum library with 6 runs. A total of 900 ng of pooled or individual peptide were separated on a reverse-phase C18 column (50 *μ*m × 150 mm, 2 *μ*m). The elution lasted 120 min with 5%–28% buffer B (0.1% formic acid in 80% acetonitrile; flow rate 0.3 μl/min). MS data were acquired using the following parameters: full-scans (m/z 350–1550) were acquired with a resolution of 60,000; PRM scans (m/z 200–2000) were run at a resolution of 30,000; retention time window was set to ± 2 min; targeted peptides were isolated using a 1.6 m/z window; 30% HCD collision energy; maximum injection time of 60 ms.

The raw pooled MS/MS data files were searched using Thermo Proteome Discover 2.1.0.81 against the SwissProt rat database (released in February 2017, containing 7,992 sequences) with precursor and fragment mass tolerances of 10 ppm and 0.02 Da, respectively. Other parameters were set as follows: trypsin digested; maximum missed cleavage sites of 2; oxidation (+15.995 Da) of methionine as a dynamic modification and carbamidomethylation (+57.021 Da) of cysteine as a static modification. The protein false discovery rate, which was determined by a target-decoy search strategy, was set to 1%^20^. Skyline software (*Version 3.6.1 10279*) was used to build spectrum library and filter peptides for PRM analysis^21^. There were 2–6 peptides of each targeted protein were selected using the following criteria: (i) digested by trypsin [KR/P] with 2 max missed cleavages, (ii) 8–18 amino acid residues, (iii) excluding the first 25 N-terminal amino acids, (iv) carbamidomethyl (C) and oxidation (M) as the structural modifications. Only unique peptides of each protein will be used for the following targeted quantitation. Retention time (RT) segment was set to 4 min for each targeted peptide with its expected RT in the center according to the pooled sample analysis^22^. Finally, there were 20 proteins with 113 peptides were scheduled. After further optimization, 20 proteins with 106 peptides were selected for validation by PRM targeted proteomics (Supplementary Table S1). The technical reproducibility of PRM assay demonstrated that there were 85 targeted peptides with their abundance CV values less than 30% (Supplementary Fig. S1).

Individual peptide samples (900 ng of each sample) were then analyzed by PRM assays. Transition settings in Skyline were as follows: precursor charges were set to +2, +3, +4, ion types with y, p, b, the product ions were set to from ion 3 to the last ion, ion match tolerance was 0.02 m/z, 6 product ions were picked, min dotp was set to 0.7. Each protein was quantitated using the summation of fragment area from its corresponding transitions. The summation of fragment area was performed by log2 transformation before statistical analysis. Differential urinary proteins were identified using a one-way ANOVA with *P*-value < 0.05.

### Functional enrichment analysis

All proteins identified to be differentially expressed between the control and Walker-256 tail-vein injection group were assigned a gene symbol using DAVID^23^ for functional annotation assessment, including biological processes, cellular components and molecular functions. The biological pathway analysis of differential proteins analyzed at four time points was performed by IPA software (Ingenuity Systems, Mountain View, CA, USA).

## Results and Discussion

### Characterization of Walker-256 tail-vein injection rats

**Figure 2:**
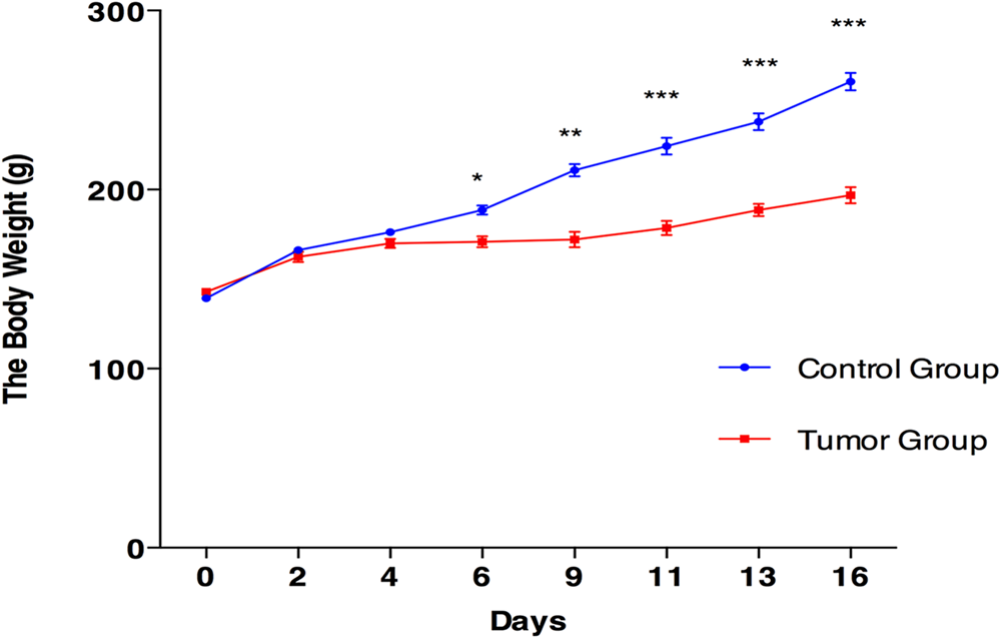
Body weight changes of Walker-256 tail-vein injection rats. The results are shown as the mean ± SD for Walker-256 lung metastatic rats and control rats. The average weight of the Walker-256 lung metastatic rats was significantly lower than that of the control rats (*p < 0.05, **p < 0.01, ***p < 0.001).

From 6 days after the tail-vein injection of Walker-256 cells, the average body weight of tail-vein injection rats was lower than that of the control rats (Fig. 2); reduced food intake was also observed in Walker-256 tail-vein injection rats. On day 9 after the tail-vein injection of Walker-256 cells, the body weight of them was significantly lower than that of the control group. Therefore, we believed that days 2 and 4 were early time points during lung tumor progression.

**Figure 3:**
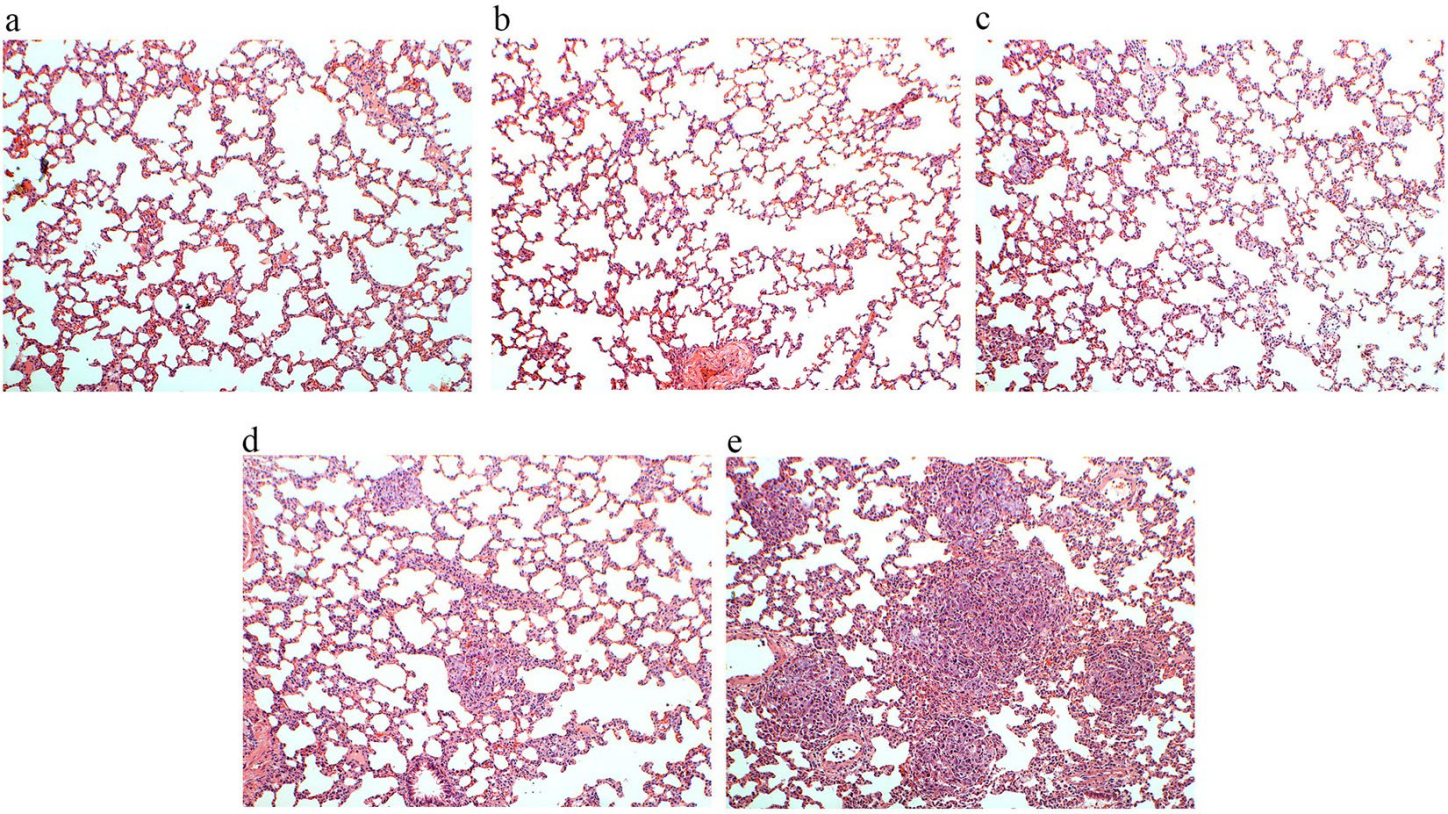
Pathological changes in Walker-256 tail-vein injection rats. The magnification was 100× for the images of H&E staining. (**a**) The control group was tail-vein injected with PBS. (**b–e**) Walker-256 lung carcinoma metastasis beginning on days 2, 4, 6, and 9 after tail-vein injection with Walker-256 tumor cells.

The pathological changes in the Walker-256 tail-vein injection rats at different time points are shown in Fig. 3. The lung tumor nodules appeared on day 4, and their number and volumes increased in the Walker-256 tail-vein injection rats during lung tumor progression. In addition, the metastatic Walker-256 cells arranged closely in Walker-256 tail-vein injection rats, and the majority of cells showed round or elliptic morphologies accompanied by poor differentiation, while their nuclei were large, irregular, and hyperchromatic. The lung tumors were scattered throughout the lung parenchyma, indicating the invasion of Walker-256 cells destroyed the lung tissue structure and the alveolar structure.

### Urine proteome changes

A total of 263 urinary proteins were identified with at least two peptides using a Triple TOF 5600 mass spectrometer, and 839 urinary proteins were identified with <1% FDR at the protein level with at least two peptides using Orbitrap Fusion Lumos mass spectrometer. All identification and quantitation details are listed in Supplementary Tables S2 and S3. Using screening criteria, there were 93 differential proteins identified using the Triple TOF 5600 and 139 differential proteins identified using Orbitrap Fusion Lumos. Details of these differential proteins at different time points by two different mass spectrometers were listed in Supplementary Tables S4 and S5.

The overlap of differential proteins identified at four time points in seven Walker-256 tail-vein injection rats are shown in a Venn diagram (Supplementary Fig. S2), and a total of 181 differential proteins were identified. Specifically, there were 62, 72, 117, and 125 differential proteins on days 2, 4, 6, and 9 after tail-vein injection of Walker-256 cells, respectively (Supplementary Table S6). All these differential proteins were used for the following functional enrichment analysis. In addition, we also identified the common differential proteins identified using two mass spectrometers on the same time points. There were 7, 16, 22, and 34 proteins that changed significantly in all 7 Walker-256 tail-vein injection rats on days 2, 4, 6, and 9, respectively. Among these commonly identified differential proteins, there were 20 urinary proteins changed significantly during the early phase (day 2 or day 4) after Walker-256 injection and continued to change at later stages (day 6 or day 9) of lung tumor progression, indicating their potential roles in early detection of lung tumor progression. These 20 differential proteins were validated by the following PRM quantification. Details are shown in Table 1.

**Table 1:** Differential urinary proteins selected for PRM validation.

UniProt ID	Protein names	ANOVA P		Trend	5600 Fold Change				Lumos Fold Change			
		5600	Lumos		D2	D4	D6	D9	D2	D4	D6	D9
O70513	Galectin–3–binding protein	0	0.0079	↑	14.58	9.55	3.24	2.69	4.39	3.61	1.97	1.86
P16391	RT1 class I histocompatibility antigen, AA alpha chain	3.91E–08	0.0053	↑	no	7.57	21.50	12.97	2.57	4.03	4.49	5.11
P02764	Alpha–1–acid glycoprotein	2.01E–09	0.0019	↑	no	2.65	6.10	10.09	no	1.91	1.95	3.72
Q6DGG1	Alpha/beta hydrolase domain–containing protein 14B	2.09E–14	< 0.00010	↑	1.90	1.51	12.49	2.21	1.97	no	4.09	no
P08649	Complement C4	6.64E–08	0.0086	↑	2.05	1.97	3.31	2.00	no	3.02	3.58	no
P15083	Polymeric immunoglobulin receptor	1.08E–08	0.035	↑	2.14	1.67	1.74	1.58	no	1.55	no	1.79
P30152	Neutrophil gelatinase–associated lipocalin	1.16E–12	< 0.00010	↑	no	2.12	no	60.14	2.26	3.21	12.42	11.84
P07314	Gamma–glutamyltranspeptidase 1	4.01E–06	0.00081	↑	no	2.99	no	1.96	1.96	1.97	no	2.01
O55006	Protein RoBo–1	1.45E–07	0.073	↑	no	2.44	2.98	2.81	no	2.00	no	2.31
B5DFC9	Nidogen–2	4.09E–08	0.038	↓	0.47	no	no	no	0.44	no	no	no
P83121	Urinary protein 3	4.72E–12	0.0023	↓	0.29	0.26	0.16	0.19	0.49	0.44	0.15	0.44
Q63041	Alpha–1–macroglobulin	7.62E–09	0.0038	↓	0.48	0.49	0.48	0.39	0.64	0.61	no	0.53
P02650	Apolipoprotein E	2.72E–14	0.0014	↓	0.44	no	0.26	0.38	0.50	no	0.38	0.54
Q9QX79	Fetuin–B	1.97E–10	0.00019	↓	no	0.17	0.14	0.07	0.64	0.61	0.37	0.41
P05544	Serine protease inhibitor A3L	1.11E–15	0.011	↓	no	0.43	0.45	0.28	0.53	0.49	0.45	0.45
P07522	Pro–epidermal growth factor	1.98E–14	0.0073	↓	0.39	0.52	0.42	0.30	0.66	no	no	0.59
P80067	Dipeptidyl peptidase 1	0.0024826	0.0018	↓	no	0.62	0.57	no	0.33	0.31	0.32	0.29
P04276	Vitamin D–binding protein	1.44E–06	0.0012	↓	no	0.38	0.43	no	0.37	0.31	0.32	0.29
P02770	Serum albumin	8.57E–08	0.0015	↓	no	0.35	no	0.33	0.57	0.51	0.62	0.64
Q5FVF9	Biotinidase	2.45E–06	0.0049	↓	no	0.60	0.53	0.49	0.61	0.56	no	0.38

### Functional enrichment analysis of differential urine proteins

**Figure 4:**
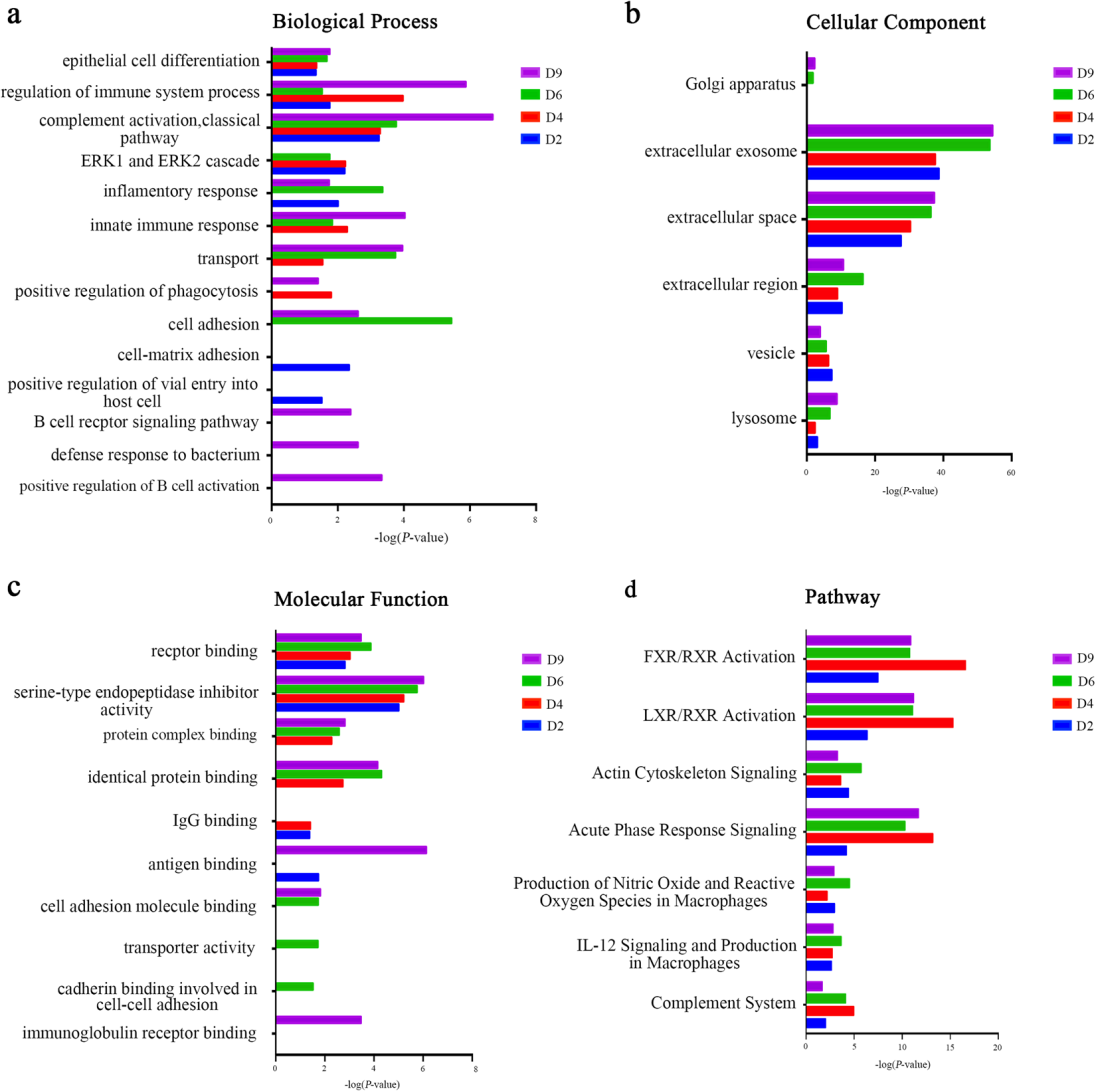
Functional analysis of differential proteins during Walker-256 lung metastatic development. Changes in biological process (**a**), cellular component (**b**), molecular function (**c**) and pathway (**d**) at four time points were classified.

The functional enrichment analysis of differential proteins was performed using DAVID^23^ and IPA. Differential proteins at four time points were classified into biological processes, cellular components, molecular functions, and pathways (Fig. 4). In the biological processes, epithelial cell differentiation, regulation of immune system processes, and the classical complement activation pathway were overrepresented on days 2, 4, 6 and 9. The ERK1 and ERK2 cascade was overrepresented on days 2, 4 and 6. The innate immune response and transport were overrepresented on days 4, 6 and 9. The cell adhesion was overrepresented on days 6 and 9. Interestingly, proteins representing the B cell receptor signaling pathway, the defense response to bacteria and the positive regulation of B cell activation appeared on day 9 (Fig. 4a).

The majority of differential proteins in the cellular component category came from the extracellular exosomes, extracellular space, cellular region, and vesicles (Fig. 4b). This result is consistent with the source of normal urine.

In the molecular function category, receptor binding and serine-type endopeptidase inhibitor activity were overrepresented at four time points, while identical protein binding, protein complex binding were overrepresented on days 4, 6, and 9. The transporter activity and cell adhesion molecule binding were both overrepresented on day 6, which is consistent with the cell adhesion and transport process protein differential expression on day 6. On day 9, immunoglobulin receptor binding was overrepresented, but its representation was still consistent with that of the B cell receptor signaling pathway and the positive regulation of B cell activation on day 9 (Fig. 4c). It is noteworthy that this molecular function did not appear before day 9.

In the pathway category, the FXR/RXR activation, LXR/RXR activation, actin cytoskeleton signaling, acute-phase response signaling, IL-12 signaling and production in macrophages, production of nitric oxide and reactive oxygen species in macrophages, and the complement system were significantly enriched during the whole metastatic progression (Fig. 4d). This result indicated that the differential proteins were indeed associated with lung tumor development.

The majority of these biological processes were reported to be associated with breast cancer metastasis or lung cancer. For example, the increasing levels of ERK1 and ERK2 were associated with breast cancer initiation, growth, and metastasis^24^. The persistent complement activation was reported for tumor cells in breast cancer, which was consistent with the timing of its overrepresentation in this study^25^. The transport and cell adhesion processes were both overrepresented on days 6 and 9, which indicated the severe metastasis during lung tumor progression. Interestingly, on day 9, proteins representing the B cell activation process became differentially expressed, indicating that a candidate antibody may be produced in this period. However, it may be too late for these antibodies to overcome Walker-256 cells and to stop the metastasis. The STRING PPI network analysis of differential proteins associated with immune system revealed a higher number of interactions than would be expected (p < 1.0 e-16) (Supplementary Table S7, Fig. S3).

### Parallel reaction monitoring (PRM) validation

After label-free quantitative analysis, there were 20 differential proteins identified with common time points since on day 2 or day 4 using two mass spectrometers for PRM quantitation. Major urinary protein was excluded for the following PRM quantitation.

**Figure 5:**
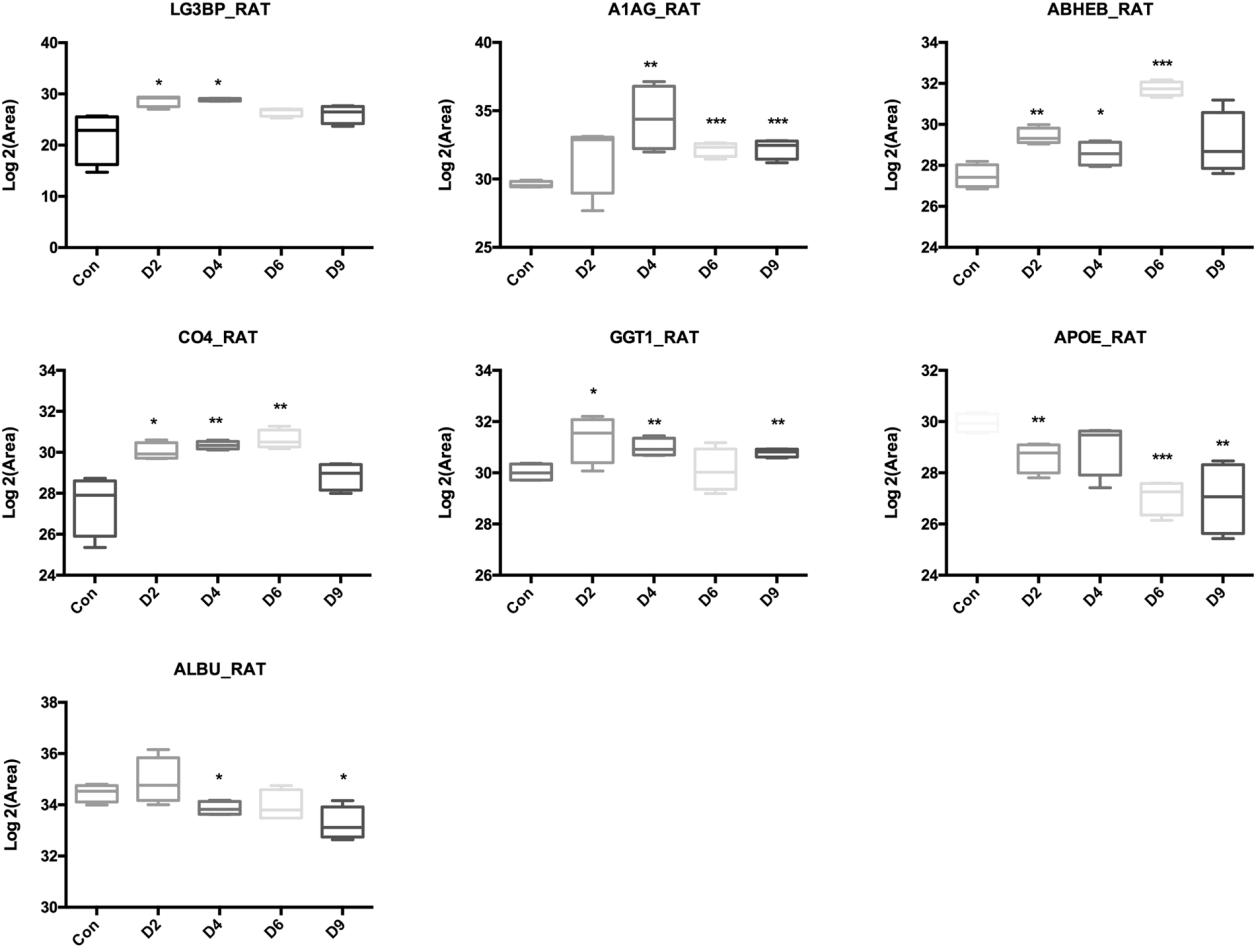
Expression of candidate urine biomarkers from Walker-256 tail-vein injection rats by PRM quantification. The x-axis represents different stages after tail-vein injection of Walker-256 tumor cells. The y-axis represents the log2 area of intensity based on PRM quantification. (*p < 0.05, **p < 0.01, ***p < 0.001).

A total of thirteen proteins were quantified statistically, showing the same expression trends with the results from the label-free quantification. Among these validated 13 proteins, 7 of them statistically changed from day 2 or day 4 with human homology. As a result, five differential proteins showed the overall upregulated trend, including LG3BP, A1AG, ABHEB, CO4, and GGT1, while two differential proteins showed the overall downregulated trend, including APOE and ALBU. These seven differential proteins showed their potential roles in early detection Walker-256 lung tumor progression (Fig. 5)

Among these seven differential proteins (LG3BP, A1AG, ABHEB, CO4, GGT1, APOE, ALBU), five of them (LG3BP, A1AG, CO4, APOE, ALBU) had been reported as lung cancer diagnostic or prognostic biomarkers, while two of them (LG3BP, GGT1) were associated with breast cancer metastasis. (1) Galectin-3-binding protein (LG3BP) was reported to be a candidate diagnostic biomarker for large-cell neuroendocrine lung carcinoma^26^. In addition, galectin-3-binding protein was also reported to induce galectin-mediated tumor cell aggregation to increase the survival of cancer cells in the bloodstream during the metastatic process^27,28^. (2) Alpha-1-acid glycoprotein (A1AG) was reported as potential lung cancer serum biomarker^29^. (3) Complement C4 (CO4) was reported as a diagnostic as well as prognostic biomarker for lung cancer^30–32^. (4) Apolipoprotein E (APOE) was reported to have diagnostic and prognostic value for nonsmall cell lung cancer in malignant pleural effusion or serum^33,34^. In addition, the expression of APOE was also reported to promote lung adenocarcinoma proliferation and migration, which are regarded as potential survival markers in lung cancer^35^. (5) The C-reactive protein/albumin ratio was reported to predict long-term outcomes of patients with operable nonsmall cell lung cancer^36^. (6) Gamma-glutamyltranspeptidase 1 (GGT1) was associated with breast cancer risk^37^. According to our results, we suggest that it is more appropriate to use a protein panel for a biomarker, as the specificities of single-protein biomarkers are not significant enough (Table 2).

**Table 2:** Details of candidate urinary biomarkers identified by PRM.

UniProt ID	Human Orthology	Protein Name	Trend	Lung Cancer Biomarkers	Breast Cancer Metastasis
O70513	Q08380	Galectin–3–binding protein (LG3BP)	↑	T^26^	^27, 28^
P02764	P02763	Alpha–1–acid glycoprotein (A1AG)	↑	S^29^	—
Q6DGG1	Q96IU4	Alpha/beta hydrolase domain–containing protein 14B (ABHEB)	↑	—	—
P08649	P0C0L4	Complement C4 (CO4)	↑	P, B1, B2^30–32^	
P07314	P19440	Gamma–glutamyltranspeptidase 1 (GGT1)	↑	—	^37^
P02650	P02649	Apolipoprotein E (APOE)	↓	M, S, T^33–35^	—
P02770	P02768	Serum albumin (ALBU)	↓	S^36^	—

T: Tissue; S: Serum; P: Plasma; B1: Bronchoalveolar lavage fluid (BALF); B2: Bronchial fluids; M: malignant pleural effusion.

We compared the differential urinary proteins between seven Walker-256 tail-vein injection rats and the subcutaneous rats^11^ (Supplementary Fig. S4). It was found that the urinary differential proteins were different when the same tumor cells grown in different rat organs. We also compared these validated seven differential proteins (LG3BP, A1AG, ABHEB, CO4, GGT1, APOE, ALBU) with early differential proteins (day 4 and day 6) in Walker-256 subcutaneous rats, GGT1 and APOE were not found in subcutaneous rat models, indicating their specific roles in detecting lung tumor progression.

### Cluster analysis of validated seven differential urinary proteins

**Figure 6:**
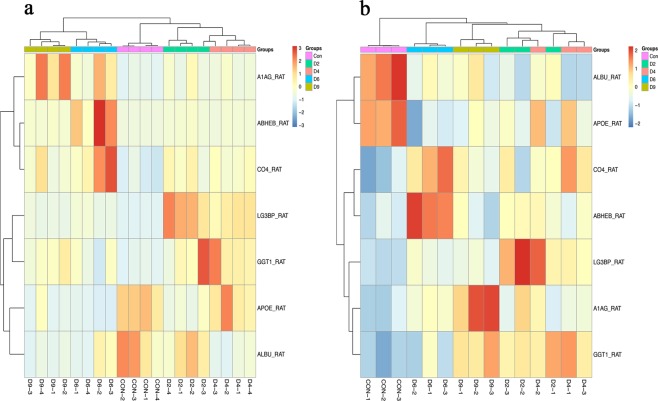
Cluster analysis of validated seven differential proteins by different mass spectrometers. Cluster analysis of differential proteins by Triple TOF 5600 (**a**) and by Orbitrap Fusion Lumos (**b**).

Clustering analysis of these validated seven differential proteins (LG3BP, A1AG, ABHEB, CO4, GGT1, APOE, ALBU) was performed in 7 Walker-256 tail-vein injection rats (Fig. 6). Samples at different lung tumor progression stages were almost clustered together using either the Triple TOF 5600 (Fig. 6a) or Orbitrap Fusion Lumos (Fig. 6b) mass spectrometer, indicating their potential roles in different stages of lung tumor progression.

In this preliminary study, only the Walker-256 tail-vein injection rat model was used for urinary biomarker discovery. Further analysis requires a large number of clinical samples to validate these candidate urinary biomarkers of lung metastasis. In addition, only common differential proteins were identified using two different mass spectrometers in this study were used for further PRM validation; other differential proteins were identified by the Triple TOF 5600 or Orbitrap Fusion Lumos cannot be ignored. Using exactly same proteomic approaches in discovery phase would be better. We still found that identifying differential proteins by two different mass spectrometers yielded differences in this study. Therefore, we propose to use the same mass spectrometers in both discovery phase and validation phase, different mass spectrometers should be considered when conducting clinical experiments.

## Conclusions

Our results indicate that (1) the urine proteome changed even on the second day after the tail-vein injection of Walker-256 cells and that (2) the urine proteome was different when the same cancer cells were grown in different tissues. Our results provide a potential panel for early detection of lung cancer.

## Supplementary information

Supplementary information.

Dataset S2 and S3.

## Data Availability

The datasets generated during the current study are available in the iProx and Figshare data repository. https://figshare.com/s/4033122b4aae7dd603b1 and https://iprox.org/page/SSV024.html;url=15630860653941bip (Password: fWhC).
