# Supplementary material for: Early urine proteome changes in the Walker-256 tail-vein injection rat model
Source: Sci Rep. 2019 Sep 24;9:13804. doi: 10.1038/s41598-019-50301-1 (PMC6760176; doi:10.1038/s41598-019-50301-1)
Supplement: Supplementary file 1 — Supplementary information. [file 41598_2019_50301_MOESM1_ESM.pdf]

Supplementary Information

Early urine proteome changes in the Walker-256 tail-vein injection rat model

Jing Wei[1], Na Ni[2], Wenshu Meng[1] & Youhe Gao[1*]

[1]Department of Biochemistry and Molecular Biology, Beijing Normal University, Gene Engineering Drug and Biotechnology Beijing Key Laboratory, Beijing, 100875, China

[2]Department of Biochemistry and Molecular Biology, College of Basic Medicine, Chongqing Medical University, Chongqing, 400016, China

[*]Corresponding author: Youhe Gao
Email: gaoyouhe@bnu.edu.cn
Phone: 86-10-5880-4382; Fax: 86-10-6521-2284

Supplementary Figure S1. The CV values of 106 PRM-targeted peptides.

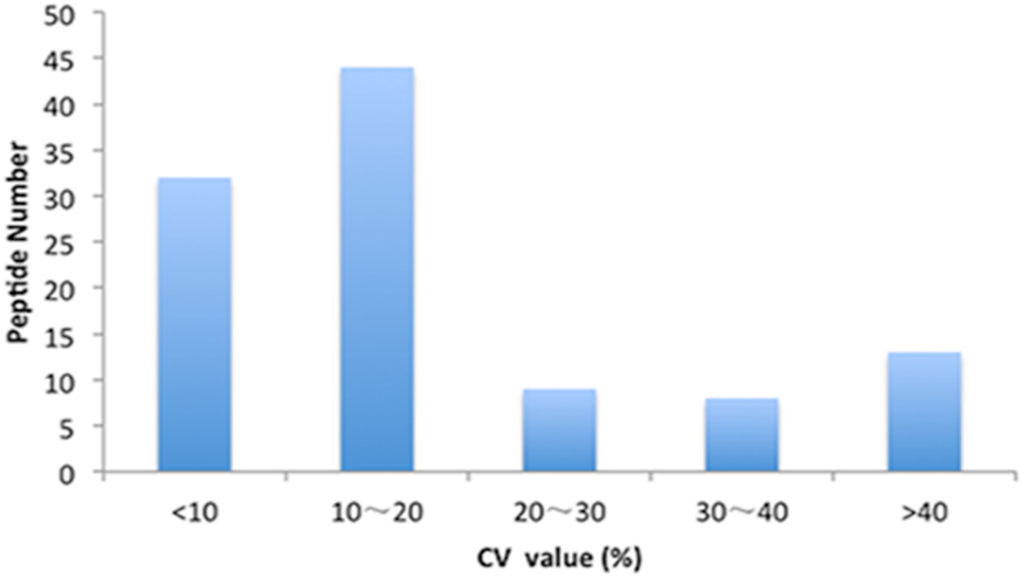

Supplementary Figure S2. Overlap of differential proteins identified in different stages in 7 Walker-256 tail-vein injection rats.

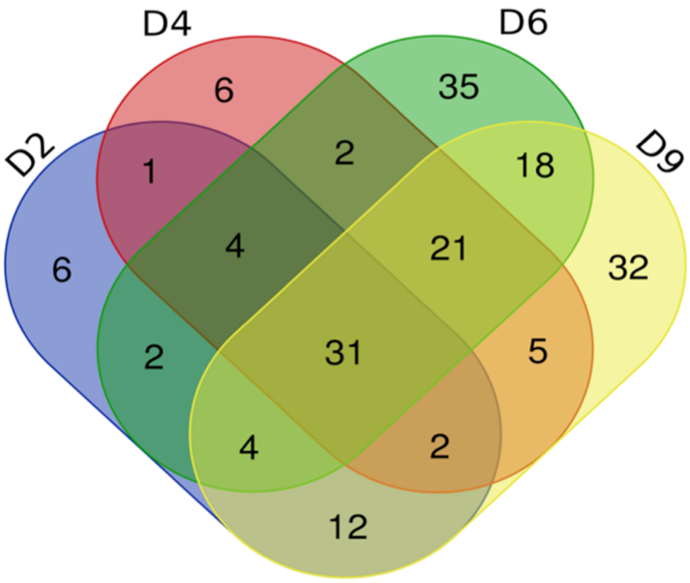

Supplementary Figure S3. STRING protein-protein interaction (PPI) network analysis of twenty proteins associated with immune system.

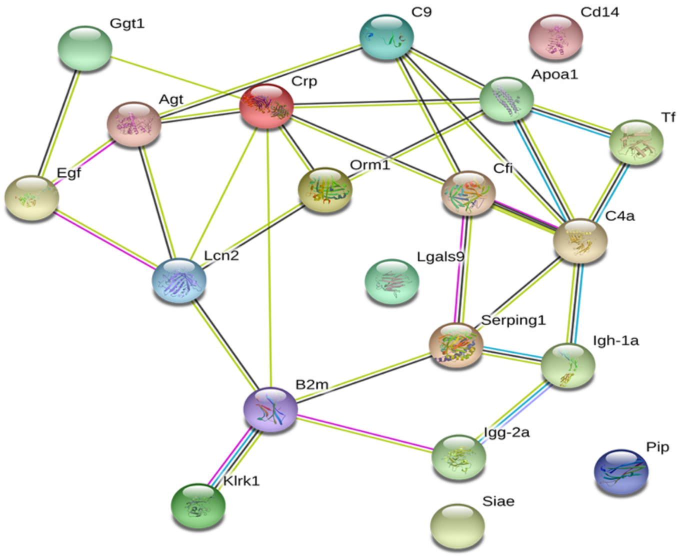

Supplementary Figure S4. Comparison of differential urinary proteins between Walker-256 tail-vein injection rats and the subcutaneous rats.

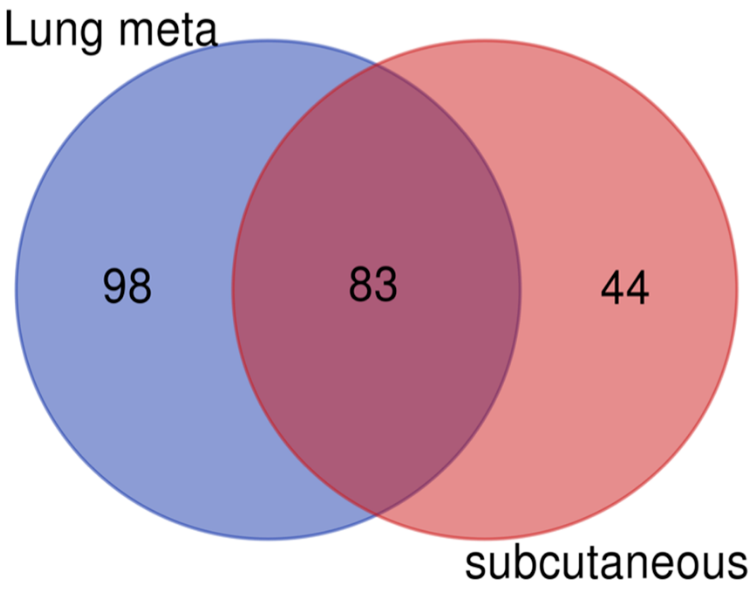

Supplementary Table S1. Retention time of 20 differential proteins with 106 peptides used for PRM validation.

m/z	z	Peptide Modified Sequence	Protein Name	t start (min)	t stop (min)	name
530.772719	2	LVNGASASEGR	sp|O70513|LG3BP_RAT	15.44	19.44	
696.029846	3	TNPEAQALWQVVGSSVIMR	sp|O70513|LG3BP_RAT	76.03	80.03	
588.281008	2	VDAEC[+57]MPVVR	sp|O70513|LG3BP_RAT	30.31	34.31	
568.310623	2	RIEVSMSSVK	sp|O70513|LG3BP_RAT	16.45	20.45	
858.904136	2	LASAYGATELQGYC[+57]GR	sp|O70513|LG3BP_RAT	40.92	44.92	
702.385039	2	SELAVSSELDLLK	sp|O70513|LG3BP_RAT	70.27	74.27	
834.860883	2	C[+57]ISFDSTGFC[+57]YVGR	sp|P83121|UP3_RAT	68.24	72.24	
783.376917	3	HIC[+57]QTYPDEIC[+57]AWVVVTTR	sp|P83121|UP3_RAT	87.44	91.44	
744.68234	3	AISYC[+57]HETMTGWVHDVLGR	sp|P80067|CATC_RAT	73.57	77.57	
491.234187	2	NWAC[+57]FVGK	sp|P80067|CATC_RAT	46.58	50.58	
763.919915	2	VYVNVAHLGGLQEK	sp|P80067|CATC_RAT	53.13	57.13	
969.77032	3	YAQDFGVVEENC[+57]FPYTATDAPC[+57]KPK	sp|P80067|CATC_RAT	70.32	74.32	
703.371735	2	DPVTGLDYWIVK	sp|P80067|CATC_RAT	88.55	92.55	
993.997376	2	GTDEC[+57]AIESIAMAAIPIPK	sp|P80067|CATC_RAT	93.49	97.49	
499.770602	2	VEPGMAPVAK	sp|Q63041|A1M_RAT	23.66	27.66	
703.869359	2	AEQGAYLGPLPYK	sp|Q63041|A1M_RAT	44.30	48.30	
527.318774	2	VAEVPALVQK	sp|Q63041|A1M_RAT	26.90	30.90	
602.832245	2	LADLPGNYITK	sp|Q63041|A1M_RAT	41.09	45.09	
580.819138	2	VNTLPLNFDK	sp|Q63041|A1M_RAT	44.50	48.50	
551.285994	2	LQDQSNIQR	sp|Q63041|A1M_RAT	9.23	13.23	
487.24614	2	GYELAHQR	sp|P07314|GGT1_RAT	9.83	13.83	

586.842947	2	LFQPSIQLAR	sp|P07314|GGT1_RAT	52.47	56.47
626.794283	2	TPALC[+57]EVFC[+57]R	sp|P07314|GGT1_RAT	48.57	52.57
749.914829	2	LADTLQILAQEGAR	sp|P07314|GGT1_RAT	61.94	65.94
531.800748	2	FVDVSQVIR	sp|P07314|GGT1_RAT	46.83	50.83
579.32987	2	NIDQVVTAGLK	sp|P07314|GGT1_RAT	40.99	44.99
800.396666	2	ELEEQLGPVAEETR	sp|P02650|APOE_RAT	39.08	43.08
484.779816	2	LGPLVEQGR	sp|P02650|APOE_RAT	20.14	24.14
620.343843	2	TANLGAGAAQPLR	sp|P02650|APOE_RAT	24.40	28.40
689.343183	2	SKMEEQTQQIR	sp|P02650|APOE_RAT	35.95	39.95
581.779687	2	MEEQTQQIR	sp|P02650|APOE_RAT	11.71	15.71
538.298373	2	LQAEIFQAR	sp|P02650|APOE_RAT	32.52	36.52
541.768591	2	DGYMLTLNR	sp|Q9QX79|FETUB_RAT	46.78	50.78
419.232502	2	SPGVPPQR	sp|Q9QX79|FETUB_RAT	12.73	16.73
485.310221	2	LVVLPFPGK	sp|Q9QX79|FETUB_RAT	55.32	59.32
694.887886	2	WFVVGLAGNAVQK	sp|P30152|NGAL_RAT	67.53	71.53
354.705588	2	VTLYGR	sp|P30152|NGAL_RAT	15.14	19.14
495.776939	2	TKGLSDELK	sp|P30152|NGAL_RAT	11.15	15.15
349.520739	3	GLSDELKER	sp|P30152|NGAL_RAT	19.61	23.61
925.914898	2	DNNIVFSVPTDQC[+57]IDN	sp|P30152|NGAL_RAT	83.30	87.30
525.727977	2	FDSDAENPR	sp|P16391|HA12_RAT	10.90	14.90
726.367515	2	TWTAADFAAQITR	sp|P16391|HA12_RAT	76.61	80.61
361.707797	2	YLELGK	sp|P16391|HA12_RAT	19.93	23.93
626.320033	2	LGQYSSPDTKR	sp|P08649|CO4_RAT	8.04	12.04
548.269478	2	LGQYSSPDTK	sp|P08649|CO4_RAT	12.33	16.33
837.781542	3	VTASEPLETLGSEGALSPGGVASLLR	sp|P08649|CO4_RAT	87.01	91.01

519.782556	2	DHAVDLIQK	sp|P08649|CO4_RAT	16.65	20.65
814.430509	2	ILSLAQEQIGDSPEK	sp|P08649|CO4_RAT	66.16	70.16
661.335349	2	IAELFSDLEER	sp|P05544|SPA3L_RAT	65.64	69.64
499.30005	2	EVLPELGIK	sp|P05544|SPA3L_RAT	46.82	50.82
639.843676	2	KVFSQQADLSR	sp|P05544|SPA3L_RAT	13.92	17.92
594.324588	2	DLYVSQVVHK	sp|P05544|SPA3L_RAT	26.42	30.42
820.04125	3	EFQTTDDQC[+57]VYNFTHLGVQR	sp|P02764|A1AG_RAT	72.28	76.28
527.344595	2	IFAHLIVLK	sp|P02764|A1AG_RAT	43.48	47.48
527.798206	2	KPDLSPELR	sp|P02764|A1AG_RAT	14.97	18.97
591.845687	2	KPDLSPELRK	sp|P02764|A1AG_RAT	9.96	13.96
481.295102	2	KIFQQAVK	sp|P02764|A1AG_RAT	7.79	11.79
623.337892	2	QQLELEKETK	sp|P02764|A1AG_RAT	11.64	15.64
427.729595	2	GQSLFFR	sp|Q6DGG1|ABHEB_RAT	28.31	32.31
390.205952	2	LAEAGYR	sp|Q6DGG1|ABHEB_RAT	10.05	14.05
541.321848	2	AVAIDLPGLGR	sp|Q6DGG1|ABHEB_RAT	56.15	60.15
660.336838	2	GYVPVAPIC[+57]TDK	sp|Q6DGG1|ABHEB_RAT	34.36	38.36
447.227416	2	INAADYAR	sp|Q6DGG1|ABHEB_RAT	15.82	19.82
807.730013	3	TPTLIVYGDQDPMGSSSFQHLK	sp|Q6DGG1|ABHEB_RAT	70.87	74.87
530.745647	2	NC[+57]QSGPLER	sp|P07522|EGF_RAT	8.70	12.70
690.883332	2	RPANILVDPTER	sp|P07522|EGF_RAT	23.06	27.06
464.758549	2	TLLEAPER	sp|P07522|EGF_RAT	21.27	25.27
729.384031	2	VVHLHAQPGTENR	sp|P07522|EGF_RAT	7.72	11.72
527.800217	2	IYFAQTALK	sp|P07522|EGF_RAT	35.80	39.80
546.282585	2	SVIEGSDLSGK	sp|P07522|EGF_RAT	19.62	23.62
526.310949	2	SLSLILYSR	sp|P04276|VTDB_RAT	55.28	59.28

573.766048	2	VC[+57]SQYAAYGK	sp|P04276|VTDB_RAT	34.81	38.81
803.435483	3	VPTANLEDVLPLAEDLTEILSR	sp|P04276|VTDB_RAT	69.31	73.31
483.766374	2	ELPEHTLK	sp|P04276|VTDB_RAT	19.89	23.89
801.878741	2	MPNASPEELADMVAK	sp|P04276|VTDB_RAT	65.93	69.93
680.287019	2	YC[+57]SSQIDAEMR	sp|P04276|VTDB_RAT	22.07	26.07
635.790252	2	SSVTFEC[+57]DLGR	sp|P15083|PIGR_RAT	34.14	38.14
681.850309	2	ETC[+57]DVIINTLGK	sp|P15083|PIGR_RAT	46.51	50.51
503.308209	2	FSVLITGLR	sp|P15083|PIGR_RAT	56.39	60.39
809.916515	2	GVTGGSVAIVC[+57]PYNPK	sp|P15083|PIGR_RAT	37.88	41.88
988.498932	2	C[+57]PVLVGTQALVQEGYEGR	sp|P15083|PIGR_RAT	62.42	66.42
488.46824	4	WSNDGC[+57]HILPSHDEGAR	sp|P15083|PIGR_RAT	26.14	30.14
538.247491	2	C[+57]PYEEHIK	sp|P02770|ALBU_RAT	8.24	12.24
575.311146	2	LVQEVTDFAK	sp|P02770|ALBU_RAT	34.72	38.72
562.252106	3	LRDNYGELADC[+57]C[+57]AK	sp|P02770|ALBU_RAT	21.41	25.41
733.393533	2	LGEYGFQNAVLVR	sp|P02770|ALBU_RAT	59.79	63.79
720.396272	2	APQVSTPTLVEAAR	sp|P02770|ALBU_RAT	33.62	37.62
567.755158	2	C[+57]C[+57]TLPEAQR	sp|P02770|ALBU_RAT	13.39	17.39
699.882998	2	SVLSPNPLELSSR	sp|Q5FVF9|BTD_RAT	51.60	55.60
918.45145	2	QNLDVYEQQVMAAAQK	sp|Q5FVF9|BTD_RAT	65.47	69.47
411.714242	2	LSC[+57]MAIK	sp|Q5FVF9|BTD_RAT	15.10	19.10
547.307352	2	MFLVANLGTK	sp|Q5FVF9|BTD_RAT	53.25	57.25
822.348671	2	QPC[+57]LGSDPGC[+57]PQDGR	sp|Q5FVF9|BTD_RAT	25.91	29.91
584.271164	2	VLSGDPYC[+57]EK	sp|Q5FVF9|BTD_RAT	18.00	22.00
702.378587	3	GC[+57]FSQLQEFAVPLLLLNR	sp|O55006|ROB1_RAT	94.66	98.66
838.389284	2	IHAMGC[+57]ATQTAC[+57]NLK	sp|O55006|ROB1_RAT	18.23	22.23

846.386742	2	IHAM[+16]GC[+57]ATQTAC[+57]NLK	sp|O55006|ROB1_RAT	16.83	20.83
520.826766	2	NVIILNNIK	sp|O55006|ROB1_RAT	36.03	40.03
694.845558	2	IDTSC[+57]VSGSPPLR	sp|O55006|ROB1_RAT	28.89	32.89
563.27476	2	YSPSLSTDQK	sp|O55006|ROB1_RAT	20.14	24.14
715.854103	2	LLPEGDDESSAAVK	sp|B5DFC9|NID2_RAT	22.67	26.67
680.85698	2	EDTSQAVLSLAAR	sp|B5DFC9|NID2_RAT	49.32	53.32
709.37534	2	ESYNVQLQLPAR	sp|B5DFC9|NID2_RAT	47.42	51.42
865.435792	2	EALYFSLTNTEQSVK	sp|B5DFC9|NID2_RAT	64.67	68.67
650.676571	3	LALDNVRPATVGGDHSTAR	sp|B5DFC9|NID2_RAT	41.66	45.66
387.185696	3	HC[+57]VPEGAPHR	sp|B5DFC9|NID2_RAT	15.25	19.25

Supplementary Table S2. Identification and quantitation details of the urine proteome identified by the Triple TOF 5600^{TM} mass spectrometer.

Supplementary Table S3. Identification and quantitation details of the urine proteome identified by the Orbitrap Fusion Lumos mass spectrometer.

Supplementary Table S4. Differential proteins at different lung carcinoma metastatic stages using the Triple TOF 5600™ mass spectrometer.

Day 2

Protein name	Accession Number	Uniprot ID	C-1-ave	C-2-ave	C-3-ave	C-4-ave	D2-1-ave	D2-2-ave	D2-3-ave	D2-4-ave	Fold change	P-value
Galectin-3-binding protein	LG3BP_RAT	O70513	6495.23	4737.04	5202.03	4671.91	79940.69	78119.47	55450.62	94189.01	14.58	1.10E-04
Dipeptidyl peptidase 4	DPP4_RAT	P14740	1955.42	3107.08	3448.45	2180.77	9126.53	11199.76	13091.26	19053.64	4.91	2.95E-03
Plasma protease C1 inhibitor	IC1_RAT	Q6P734	217.80	149.58	296.34	107.65	546.70	608.40	802.14	674.84	3.41	5.01E-04
Peroxiredoxin-6	PRDX6_RAT	O35244	190.32	254.11	176.82	419.56	893.46	695.53	993.51	850.25	3.30	3.70E-04
Ester hydrolase C11orf54 homolog	CK054_RAT	Q5U2Q3	296.05	242.06	654.70	356.42	767.62	1646.60	1307.29	1378.51	3.29	5.04E-03
Glutathione S-transferase omega-1	GSTO1_RAT	Q9Z339	85.86	23.25	55.29	72.26	112.00	241.84	230.29	140.48	3.06	1.31E-02
Aminoacylase-1A	ACY1A_RAT	Q6AYS7	85.82	152.80	146.75	253.90	364.78	341.97	719.51	271.12	2.66	4.72E-02
Heat shock cognate 71 kDa protein	HSP7C_RAT	P63018	292.17	528.70	713.52	596.39	1464.03	1256.31	1570.68	848.21	2.41	6.22E-03
Ezrin	EZRI_RAT	P31977	297.85	234.07	322.32	252.68	686.97	573.51	993.85	401.96	2.40	2.20E-02
1,2-dihydroxy-3-keto-5-methylthiopentene dioxygenase	MTND_RAT	Q562C9	543.48	517.56	536.12	620.31	893.83	1628.25	939.42	1552.68	2.26	1.20E-02
Eukaryotic translation initiation factor 6	IF6_RAT	Q3KRD8	105.59	138.76	308.05	218.57	469.23	505.62	432.50	311.10	2.23	8.65E-03

Polymeric immunoglobulin receptor	PIGR_RAT	P15083	5154.42	6163.42	4987.62	6152.82	9422.45	11066.83	11009.38	16628.11	2.14	7.24E-03
Aminopeptidase N	AMPN_RAT	P15684	5377.99	6617.70	8086.53	8134.32	14658.06	13697.58	22977.41	8582.10	2.12	4.09E-02
Complement C4	CO4_RAT	P08649	1809.03	1995.60	2790.75	1569.02	3877.50	5066.54	2978.23	4848.68	2.05	7.77E-03
Galectin-5	LEG5_RAT	P47967	1628.11	1748.44	1786.88	1586.58	3513.37	2968.45	3338.55	3395.38	1.96	1.44E-05
Protein ABHD14B	ABHEB_RAT	Q6DGG1	2563.46	2723.90	3017.87	2978.75	5325.29	7857.20	3808.12	4457.46	1.90	2.95E-02
Matrix remodeling-associated protein 8	MXRA8_RAT	Q5XI43	13448.61	11221.15	11671.83	12715.64	7186.83	7350.99	6618.66	10715.31	0.65	6.61E-03
Urinary protein 2	UP2_RAT	P81828	1422433.24	1241156.59	1336532.70	873208.70	786210.05	728893.89	807559.12	399291.65	0.56	1.29E-02
Alpha-1-macroglobulin	A1M_RAT	Q63041	41056.72	68681.06	75262.42	69051.40	31513.57	39227.72	31880.55	20175.34	0.48	8.76E-03
Prolactin-inducible protein homolog	PIP_RAT	O70417	4288.10	3568.90	4011.51	4787.17	1880.58	2874.31	1714.33	1378.70	0.47	1.72E-03
Nidogen-2	NID2_RAT	B5DFC9	131.11	289.05	272.20	194.16	127.90	127.44	76.51	85.60	0.47	2.38E-02
Apolipoprotein E	APOE_RAT	P02650	2501.84	2317.07	2350.01	1837.76	1053.41	781.66	1270.63	819.37	0.44	4.46E-04
Kallikrein-1	KLK1_RAT	P00758	109175.32	96543.58	126167.61	66923.88	33423.86	49989.03	37819.89	38917.99	0.40	3.71E-03
Pro-epidermal growth factor	EGF_RAT	P07522	205469.33	256964.18	256263.32	200314.41	80901.99	82586.61	89569.74	108385.39	0.39	1.64E-04
Urinary protein 3	UP3_RAT	P83121	597128.73	372590.31	437635.55	235249.53	81893.59	109031.28	146242.89	132467.65	0.29	8.59E-03
Anthrax toxin receptor 1	ANTR1_RAT	Q0PMD2	423.84	535.59	906.01	1637.22	110.42	116.35	178.17	300.00	0.20	4.52E-02

Day 4

Protein name	Accession Number	Uniprot ID	C-1-ave	C-2-ave	C-3-ave	C-4-ave	D4-1-ave	D4-2-ave	D4-3-ave	D4-4-ave	Fold change	P-value
Voltage-dependent anion-selective channel protein 1	VDAC1_RAT	Q9Z2L0	12.08	2.23	4.36	16.60	294.92	203.92	203.54	390.09	30.97	1.04E-03
Chloride intracellular channel protein 1	CLIC1_RAT	Q6MG61	34.72	39.08	55.08	80.95	395.75	409.92	1161.39	457.02	11.55	2.47E-02
Moesin	MOES_RAT	O35763	22.32	19.04	59.26	79.61	200.89	325.96	812.73	560.16	10.54	1.94E-02
Galectin-3-binding protein	LG3BP_RAT	O70513	6495.23	4737.04	5202.03	4671.91	56826.81	50470.39	36492.42	57692.30	9.55	9.46E-05
RT1 class I histocompatibility antigen, AA alpha chain	HA12_RAT	P16391	10.56	82.98	5.95	68.84	326.91	94.54	329.12	523.44	7.57	2.18E-02
Dipeptidyl peptidase 4	DPP4_RAT	P14740	1955.42	3107.08	3448.45	2180.77	13819.20	14910.07	15303.93	25462.24	6.50	1.71E-03
Fructose-1,6-bisphosphatase 1	F16P1_RAT	P19112	5.36	37.48	72.47	81.21	166.63	263.06	279.24	157.79	4.41	3.55E-03
Na(+)/H(+) exchange regulatory cofactor NHE-RF3	NHRF3_RAT	Q9JJ40	974.57	645.47	919.39	958.93	2464.76	3903.89	5410.83	2288.37	4.02	1.12E-02
Aminoacylase-1A	ACY1A_RAT	Q6AYS7	85.82	152.80	146.75	253.90	473.93	445.70	968.54	378.59	3.55	2.70E-02
Glutamate--cysteine ligase catalytic subunit	GSH1_RAT	P19468	270.37	352.22	439.54	388.56	1073.19	1017.30	1914.70	928.53	3.40	9.43E-03
Actin, cytoplasmic 2	ACTG_RAT	P63259	912.62	928.35	1032.83	1078.25	2600.17	2819.61	4886.52	2280.93	3.18	1.07E-02

Ezrin	EZRI_RAT	P31977	297.85	234.07	322.32	252.68	649.33	680.92	1255.72	739.03	3.00	8.49E-03
Glutathione hydrolase 1 proenzyme	GGT1_RAT	P07314	5481.38	4078.44	5259.52	4929.68	12522.48	12565.62	22270.18	11772.63	2.99	7.94E-03
Glutathione S-transferase omega-1	GSTO1_RAT	Q9Z339	85.86	23.25	55.29	72.26	105.08	129.96	286.88	177.79	2.96	3.43E-02
Neutral and basic amino acid transport protein rBAT	SLC31_RAT	Q64319	3351.77	4862.65	6876.10	6753.44	11700.29	15179.94	24655.07	11092.86	2.87	2.00E-02
Uromodulin	UROM_RAT	P27590	134251.79	92310.72	160725.97	64066.37	236806.72	478172.59	353040.49	218008.18	2.85	1.71E-02
Alpha-1-acid glycoprotein	A1AG_RAT	P02764	6942.25	2688.44	9681.74	4412.74	11474.02	22382.77	13460.87	15595.10	2.65	1.32E-02
Aminopeptidase N	AMPN_RAT	P15684	5377.99	6617.70	8086.53	8134.32	15114.49	16822.28	21977.22	17365.59	2.53	5.39E-04
Protein RoBo-1	ROB1_RAT	O55006	1406.22	1778.47	2123.98	1187.68	3803.30	4070.66	2631.82	5373.83	2.44	7.86E-03
Plasma protease C1 inhibitor	IC1_RAT	Q6P734	217.80	149.58	296.34	107.65	503.74	583.59	316.68	439.95	2.39	8.58E-03
Heat shock cognate 71 kDa protein	HSP7C_RAT	P63018	292.17	528.70	713.52	596.39	1248.52	1075.28	1588.47	1084.35	2.34	2.99E-03
Fructose-bisphosphate aldolase B	ALDOB_RAT	P00884	163.12	482.09	551.71	662.71	930.12	926.42	1226.39	897.52	2.14	7.05E-03
Neutrophil gelatinase-associated lipocalin	NGAL_RAT	P30152	934.08	1730.69	1282.76	1254.48	2613.47	4155.83	2222.15	2013.77	2.12	2.97E-02
Complement C4	CO4_RAT	P08649	1809.03	1995.60	2790.75	1569.02	3776.55	3472.07	4333.48	4462.22	1.97	1.40E-03
Glutamyl aminopeptidase	AMPE_RAT	P50123	3133.62	3821.76	2924.06	2868.03	5482.84	5531.73	7344.18	6242.66	1.93	8.90E-04
Galectin-5	LEG5_RAT	P47967	1628.11	1748.44	1786.88	1586.58	3578.90	2170.76	2797.31	3765.92	1.82	9.55E-03

Elongation factor 1-alpha 1	EF1A1_RAT	P62630	158.60	172.36	205.10	200.30	248.13	349.35	457.52	238.39	1.76	3.78E-02
Intercellular adhesion molecule 1	ICAM1_RAT	Q00238	896.79	763.94	746.70	989.22	1502.62	1383.23	1399.11	1457.33	1.69	9.26E-05
Polymeric immunoglobulin receptor	PIGR_RAT	P15083	5154.42	6163.42	4987.62	6152.82	8753.07	7555.02	9843.70	11304.25	1.67	4.72E-03
Monocyte differentiation antigen CD14	CD14_RAT	Q63691	1677.60	1313.95	1558.14	1137.99	2082.51	2364.06	1941.81	3026.19	1.66	1.35E-02
1,2-dihydroxy-3-keto-5-methylthiopentene dioxygenase	MTND_RAT	Q562C9	543.48	517.56	536.12	620.31	669.87	857.21	944.59	1037.30	1.58	7.44E-03
Protein ABHD14B	ABHEB_RAT	Q6DGG1	2563.46	2723.90	3017.87	2978.75	4249.93	5361.56	3207.21	4249.43	1.51	1.88E-02
Glandular kallikrein-7	KLK7_RAT	P36373	26508.40	25443.40	21771.96	17632.89	13194.74	17088.46	14301.89	16408.81	0.67	1.38E-02
Matrix remodeling-associated protein 8	MXRA8_RAT	Q5XI43	13448.61	11221.15	11671.83	12715.64	7609.13	8567.21	7127.43	9216.62	0.66	9.63E-04
Gamma-glutamyl hydrolase	GGH_RAT	Q62867	11958.96	9938.26	7858.29	10146.09	7200.40	6885.93	5394.30	5229.93	0.62	8.19E-03
Dipeptidyl peptidase 1	CATC_RAT	P80067	1558.48	1229.80	1431.85	1316.22	546.82	1150.78	874.37	851.20	0.62	1.00E-02
Biotinidase	BTD_RAT	Q5FVF9	1192.86	1350.53	1536.79	1617.23	878.01	733.16	666.64	1139.39	0.60	6.91E-03
Complement factor D	CFAD_RAT	P32038	23525.53	26731.90	16684.04	18204.12	12658.46	11650.39	10852.25	15590.78	0.60	1.51E-02
Prolactin-inducible protein homolog	PIP_RAT	O70417	4288.10	3568.90	4011.51	4787.17	2889.18	3110.92	1749.44	1024.91	0.53	1.19E-02

Pro-epidermal growth factor	EGF_RAT	P07522	205469.33	256964.18	256263.32	200314.41	122928.04	142527.86	98571.02	115884.09	0.52	8.88E-04
Alpha-1-macroglobulin	A1M_RAT	Q63041	41056.72	68681.06	75262.42	69051.40	33721.65	39302.82	29404.65	21509.11	0.49	8.73E-03
Alpha-2-HS-glycoprotein	FETUA_RAT	P24090	63139.98	45308.41	39227.32	23745.94	20299.45	19699.25	19622.31	23295.07	0.48	3.55E-02
Serine protease inhibitor A3K	SPA3K_RAT	P05545	136053.98	72266.76	96119.60	61995.71	38597.22	46669.11	39324.49	47889.25	0.47	2.68E-02
Calbindin	CALB1_RAT	P07171	2138.46	2135.96	2264.95	3257.98	1276.33	739.47	665.03	1928.16	0.47	1.74E-02
Carboxylesterase 1C	EST1C_RAT	P10959	710.06	1063.03	931.78	821.31	408.60	299.56	470.95	445.82	0.46	1.35E-03
Serine protease inhibitor A3L	SPA3L_RAT	P05544	143070.26	122102.83	114867.72	97155.02	41901.52	50786.54	45675.44	64656.89	0.43	6.91E-04
C-reactive protein	CRP_RAT	P48199	1325.74	2517.67	3536.33	2729.19	1155.26	1311.73	571.81	1114.46	0.41	2.18E-02
Vitamin D-binding protein	VTDB_RAT	P04276	3002.10	3110.91	5259.74	2024.71	1472.05	661.77	1158.76	1734.27	0.38	2.71E-02
Serotransferrin	TRFE_RAT	P12346	59073.76	73843.06	102271.92	32671.93	26300.04	19425.30	22024.91	29039.06	0.36	2.69E-02
Kallikrein-1	KLK1_RAT	P00758	109175.32	96543.58	126167.61	66923.88	32896.12	49302.26	29933.57	30694.79	0.36	2.98E-03
Serum albumin	ALBU_RAT	P02770	755261.22	1171882.58	1118999.67	464187.68	358136.85	222931.98	263403.09	399527.10	0.35	1.61E-02
Parvalbumin alpha	PRVA_RAT	P02625	922.54	385.40	424.59	448.22	146.83	151.30	217.34	140.68	0.30	2.45E-02
Urinary protein 3	UP3_RAT	P83121	597128.73	372590.31	437635.55	235249.53	123413.25	103796.84	134295.08	60776.64	0.26	7.37E-03
Fetuin-B	FETUB_RAT	Q9QX79	1284.70	1244.00	1593.01	361.06	261.48	67.97	116.06	315.51	0.17	1.40E-02

Day 6

Protein name	Accession Number	Uniprot ID	C-1-ave	C-2-ave	C-3-ave	C-4-ave	D6-1-ave	D6-2-ave	D6-3-ave	D6-4-ave	Fold change	P-value
Voltage-dependent anion-selective channel protein 1	VDAC1_RAT	Q9Z2L0	12.08	2.23	4.36	16.60	124.20	101.60	318.88	319.75	24.51	1.34E-02
RT1 class I histocompatibility antigen, AA alpha chain	HA12_RAT	P16391	10.56	82.98	5.95	68.84	755.01	294.70	1232.19	1337.34	21.50	1.15E-02
Superoxide dismutase [Cu-Zn]	SODC_RAT	P07632	27083.78	8448.26	11800.68	10495.25	146476.38	389967.80	336982.85	63022.72	16.19	2.98E-02
Protein ABHD14B	ABHEB_RAT	Q6DGG1	2563.46	2723.90	3017.87	2978.75	31048.85	57506.93	41777.80	10575.73	12.49	1.66E-02
Class I histocompatibility antigen, Non-RT1	HA11_RAT	P15978	659.95	600.99	616.90	550.82	5000.86	889.89	9335.43	9579.71	10.21	3.48E-02
Chloride intracellular channel protein 1	CLIC1_RAT	Q6MG61	34.72	39.08	55.08	80.95	348.24	232.30	592.06	214.03	6.61	1.53E-02
Eukaryotic translation initiation factor 6	IF6_RAT	Q3KRD8	105.59	138.76	308.05	218.57	893.04	1925.39	1855.66	380.44	6.56	3.03E-02
Moesin	MOES_RAT	O35763	22.32	19.04	59.26	79.61	250.63	206.83	208.84	464.22	6.27	9.34E-03
Alpha-1-acid glycoprotein	A1AG_RAT	P02764	6942.25	2688.44	9681.74	4412.74	19538.06	67596.98	41774.04	15728.36	6.10	4.59E-02
Fructose-1,6-bisphosphatase 1	F16P1_RAT	P19112	5.36	37.48	72.47	81.21	345.30	219.51	260.29	115.97	4.79	1.04E-02
1,2-dihydroxy-3-keto-5-methylthiopentene dioxygenase	MTND_RAT	Q562C9	543.48	517.56	536.12	620.31	1905.35	2980.55	3147.71	2283.50	4.65	4.56E-04

6-phosphogluconolactonase	6PGL_RAT	P85971	2358.34	4635.95	3884.81	2349.28	9814.13	17621.67	24608.85	6764.28	4.45	3.08E-02
Galectin-5	LEG5_RAT	P47967	1628.11	1748.44	1786.88	1586.58	4834.64	5285.71	10910.10	5680.67	3.96	1.27E-02
Dipeptidyl peptidase 4	DPP4_RAT	P14740	1955.42	3107.08	3448.45	2180.77	7143.00	6868.67	7407.21	14130.47	3.32	1.32E-02
Complement C4	CO4_RAT	P08649	1809.03	1995.60	2790.75	1569.02	3949.38	8404.87	9831.39	4863.80	3.31	1.63E-02
Galectin-3-binding protein	LG3BP_RAT	O70513	6495.23	4737.04	5202.03	4671.91	20986.51	12304.53	14048.10	21075.31	3.24	2.29E-03
Protein RoBo-1	ROB1_RAT	O55006	1406.22	1778.47	2123.98	1187.68	3843.20	6135.96	3324.94	6022.65	2.98	5.46E-03
Ezrin	EZRI_RAT	P31977	297.85	234.07	322.32	252.68	823.09	948.82	830.19	530.64	2.83	1.45E-03
Plasma protease C1 inhibitor	IC1_RAT	Q6P734	217.80	149.58	296.34	107.65	589.97	538.08	585.13	296.85	2.61	8.67E-03
Intercellular adhesion molecule 1	ICAM1_RAT	Q00238	896.79	763.94	746.70	989.22	2754.68	2274.78	2091.40	1464.74	2.53	3.15E-03
Aminoacylase-1A	ACY1A_RAT	Q6AYS7	85.82	152.80	146.75	253.90	468.52	333.16	452.31	269.41	2.38	9.68E-03
Uromodulin	UROM_RAT	P27590	134251.79	92310.72	160725.97	64066.37	278075.17	245329.77	290898.64	226925.96	2.31	1.31E-03
Keratin, type II cytoskeletal 1	K2C1_RAT	Q6IMF3	1726.96	1110.77	1024.90	1093.09	3349.41	2768.38	1835.52	2788.46	2.17	6.44E-03
Prothrombin	THRB_RAT	P18292	14151.57	12674.25	15071.46	9866.89	16506.66	27027.21	34273.28	18310.24	1.86	4.08E-02
Heat shock cognate 71 kDa protein	HSP7C_RAT	P63018	292.17	528.70	713.52	596.39	1149.99	741.62	949.16	1019.45	1.81	1.27E-02
Actin, cytoplasmic 2	ACTG_RAT	P63259	912.62	928.35	1032.83	1078.25	1857.36	2253.24	1842.87	1136.84	1.79	1.58E-02
Polymeric immunoglobulin receptor	PIGR_RAT	P15083	5154.42	6163.42	4987.62	6152.82	6665.42	11557.07	9768.56	11026.24	1.74	1.10E-02
Monocyte differentiation antigen CD14	CD14_RAT	Q63691	1677.60	1313.95	1558.14	1137.99	2616.03	2111.20	1756.00	2997.03	1.67	1.93E-02
Complement factor D	CFAD_RAT	P32038	23525.53	26731.90	16684.04	18204.12	8898.00	11148.16	15789.69	14739.81	0.59	2.23E-02

Cadherin-related family member 5	CDHR5_RAT	Q9JIK1	1823.01	1528.75	1524.30	1507.70	894.01	1174.54	849.47	787.61	0.58	1.09E-03
Dipeptidyl peptidase 1	CATC_RAT	P80067	1558.48	1229.80	1431.85	1316.22	453.10	1002.30	892.77	784.33	0.57	4.87E-03
Biotinidase	BTD_RAT	Q5FVF9	1192.86	1350.53	1536.79	1617.23	856.63	516.02	628.28	1041.39	0.53	4.62E-03
Matrix remodeling-associated protein 8	MXRA8_RAT	Q5XI43	13448.61	11221.15	11671.83	12715.64	7350.44	2871.25	5816.73	9507.55	0.52	7.43E-03
Gamma-glutamyl hydrolase	GGH_RAT	Q62867	11958.96	9938.26	7858.29	10146.09	4847.25	4495.88	5282.34	5992.68	0.52	1.72E-03
Meprin A subunit alpha	MEP1A_RAT	Q64230	21520.80	23002.06	19840.58	14924.16	11446.55	6113.45	8669.08	13421.27	0.50	5.85E-03
Alpha-1-macroglobulin	A1M_RAT	Q63041	41056.72	68681.06	75262.42	69051.40	32227.24	26526.82	30262.71	32694.06	0.48	5.31E-03
Serine protease inhibitor A3L	SPA3L_RAT	P05544	143070.26	122102.83	114867.72	97155.02	40022.45	51088.79	64757.01	60979.90	0.45	1.04E-03
Calbindin	CALB1_RAT	P07171	2138.46	2135.96	2264.95	3257.98	1241.56	1329.71	607.72	1064.87	0.43	4.55E-03
Vitamin D-binding protein	VTDB_RAT	P04276	3002.10	3110.91	5259.74	2024.71	1425.30	1725.38	1303.41	1301.06	0.43	3.23E-02
Pro-epidermal growth factor	EGF_RAT	P07522	205469.33	256964.18	256263.32	200314.41	102117.81	67538.25	83784.70	134102.21	0.42	7.48E-04
Carboxylesterase 1C	EST1C_RAT	P10959	710.06	1063.03	931.78	821.31	370.89	294.73	310.48	411.80	0.39	5.55E-04
Serine protease inhibitor A3K	SPA3K_RAT	P05545	136053.98	72266.76	96119.60	61995.71	26645.16	24747.85	39485.27	45482.73	0.37	1.55E-02
Kallikrein-1	KLK1_RAT	P00758	109175.32	96543.58	126167.61	66923.88	21848.82	34298.77	34436.21	40251.30	0.33	2.18E-03
Prolactin-inducible protein homolog	PIP_RAT	O70417	4288.10	3568.90	4011.51	4787.17	1771.23	745.42	1411.81	924.75	0.29	1.42E-04
Apolipoprotein E	APOE_RAT	P02650	2501.84	2317.07	2350.01	1837.76	706.65	493.99	495.07	646.83	0.26	3.63E-05
Alpha-1-inhibitor 3	A1I3_RAT	P14046	1003.76	2150.43	2418.99	1794.29	604.35	202.64	456.11	617.18	0.26	5.32E-03
Murinoglobulin-1	MUG1_RAT	Q03626	329.67	644.40	802.68	526.52	117.26	30.53	210.51	160.31	0.23	5.81E-03
Urinary protein 3	UP3_RAT	P83121	597128.73	372590.31	437635.55	235249.53	46169.01	42795.04	120270.47	59348.22	0.16	4.35E-03
Fetuin-B	FETUB_RAT	Q9QX79	1284.70	1244.00	1593.01	361.06	199.47	152.67	149.75	130.37	0.14	1.10E-02

Day 9

Protein name	Accession Number	Uniprot ID	C-1-ave	C-2-ave	C-3-ave	C-4-ave	D9-1-ave	D9-2-ave	D9-3-ave	D9-4-ave	Fold change	P-value
Neutrophil gelatinase-associated lipocalin	NGAL_RAT	P30152	934.08	1730.69	1282.76	1254.48	100267.88	93240.80	19657.81	99675.58	60.14	7.74E-03
Ig gamma-2B chain C region	IGG2B_RAT	P20761	49.59	140.16	108.34	76.79	1920.66	2382.62	1105.86	4399.53	26.17	1.51E-02
Moesin	MOES_RAT	O35763	22.32	19.04	59.26	79.61	784.81	1032.89	1055.79	876.23	20.81	1.04E-05
Voltage-dependent anion-selective channel protein 1	VDAC1_RAT	Q9Z2L0	12.08	2.23	4.36	16.60	111.14	161.84	123.23	261.25	18.64	3.91E-03
RT1 class I histocompatibility antigen, AA alpha chain	HA12_RAT	P16391	10.56	82.98	5.95	68.84	555.18	673.18	806.41	147.58	12.97	1.27E-02
Chloride intracellular channel protein 1	CLIC1_RAT	Q6MG61	34.72	39.08	55.08	80.95	430.88	817.45	411.89	523.65	10.41	1.95E-03
Alpha-1-acid glycoprotein	A1AG_RAT	P02764	6942.25	2688.44	9681.74	4412.74	48431.02	82608.22	24282.24	84007.36	10.09	9.90E-03
Ester hydrolase C11orf54 homolog	CK054_RAT	Q5U2Q3	296.05	242.06	654.70	356.42	2929.30	5121.04	1831.68	5714.24	10.07	8.69E-03
Ig lambda-2 chain C region	LAC2_RAT	P20767	22397.05	42535.19	50833.92	48642.66	690166.74	202432.98	153519.33	322266.88	8.32	4.80E-02
Peroxiredoxin-6	PRDX6_RAT	O35244	190.32	254.11	176.82	419.56	815.21	3398.85	1428.99	1906.10	7.25	2.60E-02
Ig kappa chain C region, B allele	KACB_RAT	P01835	781.45	76511.14	50950.53	96490.70	686469.74	293967.98	214765.14	407419.21	7.13	1.69E-02
Carbonic anhydrase 3	CAH3_RAT	P14141	100.63	180.17	230.76	274.60	2071.51	1076.27	774.95	1035.64	6.31	1.10E-02

Fructose-1,6-bisphosphatase 1	F16P1_RAT	P19112	5.36	37.48	72.47	81.21	404.58	241.78	135.38	395.18	5.99	1.07E-02
Ig kappa chain V region S211	KVX01_RAT	P01681	1399.47	1135.12	1190.59	1308.76	12322.10	3875.08	2919.89	7875.02	5.36	4.31E-02
Ig gamma-2A chain C region	IGG2A_RAT	P20760	1610.46	1052.48	1316.68	974.07	6965.21	6175.45	2321.32	7798.08	4.70	9.47E-03
Dipeptidyl peptidase 4	DPP4_RAT	P14740	1955.42	3107.08	3448.45	2180.77	7604.03	11804.11	9630.61	11526.64	3.79	3.65E-04
Na(+)/H(+) exchange regulatory cofactor NHE-RF1	NHRF1_RAT	Q9JJ19	1152.88	343.64	873.70	501.33	1801.60	3875.69	1684.76	3150.10	3.66	1.46E-02
Na(+)/H(+) exchange regulatory cofactor NHE-RF3	NHRF3_RAT	Q9JJ40	974.57	645.47	919.39	958.93	2854.30	3420.30	2007.38	4191.61	3.57	2.99E-03
Cathepsin S	CATS_RAT	Q02765	833.03	797.42	650.10	1671.21	3719.22	3476.16	3005.57	3814.24	3.55	1.38E-04
Plasma protease C1 inhibitor	IC1_RAT	Q6P734	217.80	149.58	296.34	107.65	527.61	770.08	688.96	651.89	3.42	3.75E-04
Actin, cytoplasmic 2	ACTG_RAT	P63259	912.62	928.35	1032.83	1078.25	3173.19	3494.49	3906.11	2668.38	3.35	1.21E-04
Superoxide dismutase [Cu-Zn]	SODC_RAT	P07632	27083.78	8448.26	11800.68	10495.25	45635.17	45853.92	34399.04	53065.75	3.09	1.89E-03
Isopentenyl-diphosphate Delta-isomerase 1	IDI1_RAT	O35760	12.18	21.91	31.39	14.25	65.80	53.18	58.16	50.83	2.86	5.05E-04
Protein RoBo-1	ROB1_RAT	O55006	1406.22	1778.47	2123.98	1187.68	3297.17	4009.11	4115.01	6833.27	2.81	1.07E-02
Ezrin	EZRI_RAT	P31977	297.85	234.07	322.32	252.68	548.45	1086.78	699.54	732.01	2.77	5.48E-03
Galectin-3-binding protein	LG3BP_RAT	O70513	6495.23	4737.04	5202.03	4671.91	13103.45	12517.30	18361.52	12803.73	2.69	8.65E-04

Keratin, type II cytoskeletal 1	K2C1_RAT	Q6IMF3	1726.96	1110.77	1024.90	1093.09	3759.53	3390.89	2572.78	3129.39	2.59	5.75E-04
Eukaryotic translation initiation factor 6	IF6_RAT	Q3KRD8	105.59	138.76	308.05	218.57	496.93	526.00	440.68	514.47	2.57	8.37E-04
Aminoacylase-1A	ACY1A_RAT	Q6AYS7	85.82	152.80	146.75	253.90	315.08	419.83	433.70	399.23	2.45	1.83E-03
Nuclear transport factor 2	NTF2_RAT	P61972	318.52	351.86	279.76	226.52	1031.93	644.67	556.88	430.97	2.26	3.07E-02
Glutamate--cysteine ligase catalytic subunit	GSH1_RAT	P19468	270.37	352.22	439.54	388.56	717.78	914.50	677.23	920.70	2.23	9.09E-04
Protein ABHD14B	ABHEB_RAT	Q6DGG1	2563.46	2723.90	3017.87	2978.75	7138.45	5359.08	6610.38	5816.89	2.21	1.69E-04
Heat shock cognate 71 kDa protein	HSP7C_RAT	P63018	292.17	528.70	713.52	596.39	997.76	1150.69	1126.25	1368.93	2.18	1.75E-03
Complement C4	CO4_RAT	P08649	1809.03	1995.60	2790.75	1569.02	2972.69	3535.73	3618.15	6198.46	2.00	3.76E-02
Monocyte differentiation antigen CD14	CD14_RAT	Q63691	1677.60	1313.95	1558.14	1137.99	2218.16	2483.38	2404.87	4069.74	1.96	2.17E-02
Glutathione hydrolase 1 proenzyme	GGT1_RAT	P07314	5481.38	4078.44	5259.52	4929.68	9210.18	13274.63	7267.09	8902.94	1.96	1.14E-02
Neutral and basic amino acid transport protein rBAT	SLC31_RAT	Q64319	3351.77	4862.65	6876.10	6753.44	10094.11	14130.81	8504.10	9610.98	1.94	1.37E-02
1,2-dihydroxy-3-keto-5-methylthiopentene dioxygenase	MTND_RAT	Q562C9	543.48	517.56	536.12	620.31	740.66	1052.35	1137.81	1012.46	1.78	2.82E-03
Intercellular adhesion molecule 1	ICAM1_RAT	Q00238	896.79	763.94	746.70	989.22	1460.87	1244.53	1308.77	1507.44	1.63	7.58E-04

Polymeric immunoglobulin receptor	PIGR_RAT	P15083	5154.42	6163.42	4987.62	6152.82	7957.51	9674.13	9028.05	8819.31	1.58	4.73E-04
Protein/nucleic acid deglycase DJ-1	PARK7_RAT	O88767	198.14	251.05	283.61	231.67	372.93	326.05	417.85	387.09	1.56	2.11E-03
Phosphatidylethanolamine-binding protein 1	PEBP1_RAT	P31044	561.97	597.09	841.43	646.53	1167.17	960.34	1084.07	911.98	1.56	4.92E-03
Aminopeptidase N	AMPN_RAT	P15684	5377.99	6617.70	8086.53	8134.32	9868.21	13874.99	8797.72	10502.67	1.53	2.74E-02
CD48 antigen	CD48_RAT	P10252	5855.87	9456.36	5330.70	6624.84	4648.73	3514.90	4947.28	4598.57	0.65	4.91E-02
Prothrombin	THRB_RAT	P18292	14151.57	12674.25	15071.46	9866.89	7992.13	7907.35	8118.10	9274.61	0.64	7.91E-03
Putative phospholipase B-like 2	PLBL2_RAT	Q4QQW8	270.61	371.29	297.49	322.57	141.94	230.83	176.85	219.27	0.61	5.88E-03
Cadherin-related family member 5	CDHR5_RAT	Q9JIK1	1823.01	1528.75	1524.30	1507.70	624.91	1180.95	1005.10	959.07	0.59	3.29E-03
Sialate O-acetylesterase	SIAE_RAT	P82450	536.61	566.99	424.39	454.93	292.80	402.58	276.94	197.52	0.59	9.29E-03
Meprin A subunit alpha	MEP1A_RAT	Q64230	21520.80	23002.06	19840.58	14924.16	10692.54	10445.42	11656.94	13581.51	0.58	4.86E-03
Matrix remodeling-associated protein 8	MXRA8_RAT	Q5XI43	13448.61	11221.15	11671.83	12715.64	6985.04	5034.07	9450.39	5772.74	0.56	2.45E-03
Choline transporter-like protein 4	CTL4_RAT	Q6MG71	859.66	523.07	611.69	418.55	222.10	301.35	367.57	313.25	0.50	2.23E-02
Glandular kallikrein-7, submandibular/renal	KLK7_RAT	P36373	26508.40	25443.40	21771.96	17632.89	9997.91	11227.99	10347.48	13888.49	0.50	1.96E-03
Fatty acid-binding protein, brain	FABP7_RAT	P55051	11861.09	11774.35	8476.35	7715.40	4983.67	4257.74	3048.11	7398.16	0.49	1.22E-02
Biotinidase	BTD_RAT	Q5FVF9	1192.86	1350.53	1536.79	1617.23	859.71	527.62	1055.40	368.36	0.49	7.53E-03
Apolipoprotein A-IV	APOA4_RAT	P02651	35825.40	69563.91	53286.01	74613.03	29683.09	25344.69	22630.05	33228.64	0.48	1.50E-02

Carboxylesterase 1C	EST1C_RAT	P10959	710.06	1063.03	931.78	821.31	361.50	369.60	407.06	478.25	0.46	9.99E-04
Low-density lipoprotein receptor-related protein 2	LRP2_RAT	P98158	19807.02	46295.08	40558.47	32906.14	13935.32	15994.91	13354.82	18033.10	0.44	1.53E-02
Corticosteroid-binding globulin	CBG_RAT	P31211	8401.20	4452.49	5832.69	7495.63	3119.42	2608.98	2846.14	2857.72	0.44	5.85E-03
Cathepsin L1	CATL1_RAT	P07154	844.76	1203.85	1082.40	821.67	321.22	393.77	543.71	395.12	0.42	1.48E-03
Lysosomal alpha-glucosidase	LYAG_RAT	Q6P7A9	1092.02	2264.26	1966.05	1276.37	788.69	575.23	836.44	372.45	0.39	1.49E-02
Gamma-glutamyl hydrolase	GGH_RAT	Q62867	11958.96	9938.26	7858.29	10146.09	3759.44	4519.91	3271.35	3871.28	0.39	4.32E-04
Alpha-1-macroglobulin	A1M_RAT	Q63041	41056.72	68681.06	75262.42	69051.40	28409.45	26198.22	16752.80	26506.35	0.39	2.89E-03
Apolipoprotein E	APOE_RAT	P02650	2501.84	2317.07	2350.01	1837.76	669.47	728.29	672.81	1320.88	0.38	5.98E-04
Serine protease inhibitor A3K	SPA3K_RAT	P05545	136053.98	72266.76	96119.60	61995.71	27144.28	37095.54	35234.15	35721.93	0.37	1.31E-02
Fibronectin	FINC_RAT	P04937	22856.12	18705.32	21644.26	18210.11	7897.20	7388.69	7788.53	6888.14	0.37	3.05E-05
Parvalbumin alpha	PRVA_RAT	P02625	922.54	385.40	424.59	448.22	186.48	236.81	167.91	203.83	0.36	3.46E-02
Alpha-2-HS-glycoprotein	FETUA_RAT	P24090	63139.98	45308.41	39227.32	23745.94	16582.75	14722.10	12250.28	16549.83	0.35	1.47E-02
Serum albumin	ALBU_RAT	P02770	755261.22	1171882.58	1118999.67	464187.68	276630.91	321743.10	228003.28	331545.78	0.33	1.27E-02
Kallikrein-1	KLK1_RAT	P00758	109175.32	96543.58	126167.61	66923.88	30605.51	30600.12	26669.21	41665.94	0.32	1.99E-03
C-reactive protein	CRP_RAT	P48199	1325.74	2517.67	3536.33	2729.19	596.64	1048.99	706.64	913.92	0.32	1.06E-02
Pro-epidermal growth factor	EGF_RAT	P07522	205469.33	256964.18	256263.32	200314.41	77696.33	65493.16	53727.95	78254.82	0.30	6.90E-05
Serine protease inhibitor A3L	SPA3L_RAT	P05544	143070.26	122102.83	114867.72	97155.02	30818.75	34891.24	36585.93	32308.75	0.28	1.10E-04

Prolactin-inducible protein homolog	PIP_RAT	O70417	4288.10	3568.90	4011.51	4787.17	853.10	1162.50	248.04	2111.20	0.26	5.80E-04
Alpha-1-inhibitor 3	A1I3_RAT	P14046	1003.76	2150.43	2418.99	1794.29	496.57	404.25	599.96	421.87	0.26	4.65E-03
Murinoglobulin-1	MUG1_RAT	Q03626	329.67	644.40	802.68	526.52	152.91	161.03	92.32	81.27	0.21	4.27E-03
Calbindin	CALB1_RAT	P07171	2138.46	2135.96	2264.95	3257.98	487.50	355.20	759.29	461.05	0.21	4.97E-04
Urinary protein 3	UP3_RAT	P83121	597128.73	372590.31	437635.55	235249.53	103345.12	89775.89	63860.14	62994.70	0.19	4.75E-03
Fetuin-B	FETUB_RAT	Q9QX79	1284.70	1244.00	1593.01	361.06	134.05	13.15	89.59	72.47	0.07	7.79E-03

Supplementary Table S5. Differential proteins at different lung carcinoma metastatic stages using the Orbitrap Fusion Lumos mass spectrometer.

Day 2

Protein name	Accession Number	Uniprot ID	C-1-ave	C-2-ave	C-3-ave	D2-1-ave	D2-2-ave	D2-3-ave	Fold change	P-value
Transforming protein RhoA	RHOA_RAT	P61589	0	2	0	7.5	4	4.5	8.00	2.19E-02
Pancreatic secretory granule membrane major glycoprotein GP2	GP2_RAT	P19218	0.5	5.5	0.5	6.5	16	11.5	5.23	4.61E-02
Galectin-3-binding protein	LG3BP_RAT	O70513	14	11.5	13	38	76	55	4.39	1.68E-02
Na(+)/H(+) exchange regulatory cofactor NHE-RF1	NHRF1_RAT	Q9JJ19	8	4.5	10.5	28.5	30.5	27.5	3.76	4.09E-04
Peptidyl-prolyl cis-trans isomerase A	PPIA_RAT	P10111	2.5	3.5	2.5	9.5	8.5	12	3.53	2.80E-03
Uromodulin	UROM_RAT	P27590	83	56.5	81	160	332.5	232.5	3.29	2.95E-02
Class I histocompatibility antigen, Non-RT1.A alpha-1 chain	HA11_RAT	P15978	5.5	6	5	11	20.5	22.5	3.27	2.46E-02
Brain acid soluble protein 1	BASP1_RAT	Q05175	2.5	0.5	1.5	6	5	3.5	3.22	2.29E-02
RT1 class I histocompatibility antigen, AA alpha chain	HA12_RAT	P16391	5.5	8	4	9	17.5	18.5	2.57	4.70E-02
Neutrophil gelatinase-associated lipocalin	NGAL_RAT	P30152	1.5	4	4	9	7.5	5	2.26	4.93E-02
Thioredoxin	THIO_RAT	P11232	4.5	2.5	4.5	6	8.5	11.5	2.26	4.86E-02

Alpha/beta hydrolase domain-containing protein 14B	ABHEB_RAT	Q6DGG1	4	7.5	6	10	12.5	12	1.97	1.11E-02
Gamma-glutamyltranspeptidase 1	GGT1_RAT	P07314	16.5	13	16.5	34	29	27	1.96	3.55E-03
Alpha-enolase	ENOA_RAT	P04764	9.5	11.5	11.5	19.5	21	15.5	1.72	1.15E-02
Galectin-9	LEG9_RAT	P97840	4.5	3	4	5	6	7	1.57	4.06E-02
Pro-epidermal growth factor	EGF_RAT	P07522	128	149.5	144.5	75.5	108	93.5	0.66	1.34E-02
Gelsolin	GELS_RAT	Q68FP1	29.5	30.5	24.5	20	16	19	0.65	1.13E-02
Alpha-1-macroglobulin	A1M_RAT	Q63041	97	124.5	130.5	75.5	78	73.5	0.64	1.60E-02
Fetuin-B	FETUB_RAT	Q9QX79	20	21.5	18	9.5	12	16.5	0.64	3.50E-02
Biotinidase	BTD_RAT	Q5FVF9	12.5	13.5	12.5	8	6.5	9	0.61	3.33E-03
Sialidase-1	NEUR1_RAT	Q99PW3	7	8	6.5	3.5	5.5	4	0.60	1.91E-02
Lysosomal thioesterase PPT2	PPT2_RAT	O70489	5.5	6.5	5	4.5	2.5	3	0.59	3.52E-02
Ig kappa chain C region, A allele	KACA_RAT	P01836	112.5	100.5	74	71	50	46.5	0.58	4.39E-02
Nuclear transport factor 2	NTF2_RAT	P61972	4.5	4.5	4	3	2.5	2	0.58	5.33E-03
Serum albumin	ALBU_RAT	P02770	257	278.5	308	191	129	160	0.57	6.43E-03
Nidogen-1 (Fragment)	NID1_RAT	P08460	11.5	12	10.5	9	3	6	0.53	4.06E-02
Serine protease inhibitor A3L	SPA3L_RAT	P05544	159.5	145.5	116	59	69.5	93.5	0.53	1.55E-02
Angiotensinogen	ANGT_RAT	P01015	8.5	8.5	10	7.5	3.5	3	0.52	4.54E-02
Cluster of Carboxylesterase 1C	EST1C_RAT [4]	P10959	14	14	16.5	10.5	4.5	8	0.52	2.06E-02
Multiple inositol polyphosphate phosphatase 1	MINP1_RAT	O35217	10.5	12.5	11.5	6	2.5	9	0.51	4.48E-02
Attractin	ATRN_RAT	Q99J86	10.5	14	13	4	8	7	0.51	1.79E-02

Apolipoprotein E	APOE_RAT	P02650	18	17.5	20.5	7.5	9	11.5	0.50	3.32E-03
Urinary protein 3	UP3_RAT	P83121	98.5	89	89	32	77	26.5	0.49	4.49E-02
Protein disulfide-isomerase	PDIA1_RAT	P04785	7	5	5	3	1.5	3.5	0.47	2.88E-02
Nidogen-2	NID2_RAT	B5DFC9	5	9	8.5	4	2	4	0.44	4.30E-02
Neogenin (Fragment)	NEO1_RAT	P97603	4.5	5.5	5	2	1.5	3	0.43	5.78E-03
Procollagen C-endopeptidase enhancer 1	PCOC1_RAT	O08628	3	5	5	2	1.5	1.5	0.38	1.78E-02
Vitamin D-binding protein	VTDB_RAT	P04276	42	42.5	51	23.5	5	21	0.37	1.15E-02
Activin receptor type-1B	ACV1B_RAT	P80202	6	4	3	1	2.5	1	0.35	4.91E-02
Dipeptidyl peptidase 1	CATC_RAT	P80067	12.5	15	8.5	4.5	4	3.5	0.33	1.39E-02
Retinol-binding protein 4	RET4_RAT	P04916	5.5	3.5	5	1	1.5	1.5	0.29	5.90E-03
Growth arrest-specific protein 6	GAS6_RAT	Q63772	6.5	7.5	6	2	1.5	1.5	0.25	4.47E-04
Apolipoprotein A-IV	APOA4_RAT	P02651	6	8.5	9	0.5	0.5	1.5	0.11	2.08E-03

Day 4

Protein name	Accession Number	Uniprot ID	C-1-ave	C-2-ave	C-3-ave	D4-1-ave	D4-2-ave	D4-3-ave	Fold change	P-value
NKG2-D type II integral membrane protein	NKG2D_RAT	O70215	0	1.5	0	4	3.5	6.5	9.33	1.68E-02
Class I histocompatibility antigen, Non-RT1.A alpha-1 chain	HA11_RAT	P15978	5.5	6	5	21.5	38.5	32	5.58	7.12E-03
N-acyl-aromatic-L-amino acid amidohydrolase (carboxylate-forming)	ACY3_RAT	Q5M876	1	1.5	2	8.5	3	7.5	4.22	4.80E-02
RT1 class I histocompatibility antigen, AA alpha chain	HA12_RAT	P16391	5.5	8	4	13.5	29	28	4.03	2.64E-02
Galectin-3-binding protein	LG3BP_RAT	O70513	14	11.5	13	38.5	67.5	33	3.61	3.54E-02
Neutrophil gelatinase-associated lipocalin	NGAL_RAT	P30152	1.5	4	4	10.5	12.5	7.5	3.21	1.39E-02
Rho GDP-dissociation inhibitor 1	GDIR1_RAT	Q5XI73	1	3	0.5	5.5	4.5	4	3.11	2.29E-02
Complement C4	CO4_RAT	P08649	4.5	7.5	14.5	34	20	26	3.02	2.38E-02
G-protein coupled receptor family C group 5 member C	GPC5C_RAT	Q3KRC4	1	3.5	4	9.5	6.5	6	2.59	3.49E-02
Neprilysin	NEP_RAT	P07861	13	12.5	16	42.5	23.5	38	2.51	2.34E-02
Thioredoxin	THIO_RAT	P11232	4.5	2.5	4.5	7.5	8	9	2.13	5.61E-03
Protein RoBo-1	ROB1_RAT	O55006	5.5	4.5	4.5	9	10	10	2.00	5.10E-04
Gamma-glutamyltranspeptidase 1	GGT1_RAT	P07314	16.5	13	16.5	35	27.5	28	1.97	5.26E-03
Alpha-1-acid glycoprotein	A1AG_RAT	P02764	14	13	22	27	37.5	29	1.91	2.60E-02

Polymeric immunoglobulin receptor	PIGR_RAT	P15083	28.5	31.5	32	36	55	52	1.55	4.71E-02
Lysosomal thioesterase PPT2	PPT2_RAT	O70489	5.5	6.5	5	3.5	4	3	0.62	1.47E-02
Attractin	ATRN_RAT	Q99J86	10.5	14	13	8	7.5	7.5	0.61	1.01E-02
Alpha-1-macroglobulin	A1M_RAT	Q63041	97	124.5	130.5	77	71	67.5	0.61	1.30E-02
Fetuin-B	FETUB_RAT	Q9QX79	20	21.5	18	11	11.5	13.5	0.61	3.50E-03
Growth arrest-specific protein 6	GAS6_RAT	Q63772	6.5	7.5	6	3.5	4	4.5	0.60	7.18E-03
Biotinidase	BTD_RAT	Q5FVF9	12.5	13.5	12.5	6	5.5	10	0.56	1.79E-02
Serum albumin	ALBU_RAT	P02770	257	278.5	308	126.5	179.5	127.5	0.51	3.97E-03
Serine protease inhibitor A3L	SPA3L_RAT	P05544	159.5	145.5	116	36.5	95.5	73.5	0.49	2.87E-02
Urinary protein 3	UP3_RAT	P83121	98.5	89	89	19	55	48	0.44	1.09E-02
Beta-2-glycoprotein 1	APOH_RAT	P26644	14.5	10	13	3.5	4	8	0.41	1.96E-02
Cluster of Carboxylesterase 1C	EST1C_RAT [4]	P10959	14	14	16.5	4.5	3	10	0.39	1.70E-02
Urinary protein 2	UP2_RAT	P81828	132	104.5	105	21.5	54.5	52	0.37	7.00E-03
Vitamin D-binding protein	VTDB_RAT	P04276	42	42.5	51	15	15.5	14	0.33	5.07E-04
Retinol-binding protein 4	RET4_RAT	P04916	5.5	3.5	5	0.5	2	2	0.32	1.55E-02
Dipeptidyl peptidase 1	CATC_RAT	P80067	12.5	15	8.5	5	2.5	3.5	0.31	1.47E-02
Cluster of Alpha-1-inhibitor 3	A1I3_RAT [4]	P14046	54.5	84.5	100.5	32	13.5	26	0.30	1.83E-02
Transthyretin	TTHY_RAT	P02767	9.5	6.5	8.5	1.5	2.5	3	0.29	4.09E-03
Beta-defensin 50	DFB50_RAT	Q30KJ2	5	4	4.5	0	1.5	0.5	0.15	1.90E-03
Apolipoprotein A-IV	APOA4_RAT	P02651	6	8.5	9	0	1.5	0.5	0.09	2.22E-03

Day 6

Protein name	Accession Number	Uniprot ID	C-1-ave	C-2-ave	C-3-ave	D6-1-ave	D6-2-ave	D6-3-ave	Fold change	P-value
Secernin-2	SCRN2_RAT	Q6AYR8	0	0	0	6.5	12.5	10	#DIV/0!	5.14E-03
Fatty acid-binding protein, brain	FABP7_RAT	P55051	0	0	0	9.5	5.5	10	#DIV/0!	4.25E-03
Isopentenyl-diphosphate Delta-isomerase 1	IDI1_RAT	O35760	0	0	0	4	6	6.5	#DIV/0!	1.97E-03
Cystatin-B	CYTB_RAT	P01041	0	0	0	5.5	6.5	3.5	#DIV/0!	4.24E-03
Acyl-protein thioesterase 1	LYPA1_RAT	P70470	0	0	0	5.5	3.5	8	#DIV/0!	1.21E-02
Probable cytosolic iron-sulfur protein assembly protein CIAO1	CIAO1_RAT	Q5M7T1	0	0	0	4.5	6.5	2.5	#DIV/0!	1.76E-02
UPF0587 protein C1orf123 homolog	CA123_RAT	Q498R7	0	0	0	4.5	4.5	6	#DIV/0!	5.62E-04
Carbonic anhydrase 1	CAH1_RAT	B0BNN3	0	0	0	2	4	7.5	#DIV/0!	4.88E-02
3-mercaptopyruvate sulfurtransferase	THTM_RAT	P97532	0	0.5	0	8	12.5	12	65.00	1.74E-03
Prostaglandin reductase 2	PTGR2_RAT	Q5BK81	0.5	0.5	0	10	10	10.5	30.50	1.97E-06
Chloride intracellular channel protein 4	CLIC4_RAT	Q9Z0W7	0	0	1	6.5	4.5	5.5	16.50	1.49E-03
ATP synthase subunit beta, mitochondrial	ATPB_RAT	P10719	0	1	0	5.5	2.5	7.5	15.50	3.16E-02
Retinol-binding protein 1	RET1_RAT	P02696	0	1	0.5	6.5	6	8.5	14.00	1.35E-03
Neutrophil gelatinase-associated lipocalin	NGAL_RAT	P30152	1.5	4	4	42.5	26	49.5	12.42	6.72E-03

NKG2-D type II integral membrane protein	NKG2D_RAT	O70215	0	1.5	0	4.5	5	4.5	9.33	1.38E-03
Ectonucleotide pyrophosphatase/phosphodiesterase family member 3	ENPP3_RAT	P97675	0	0	2	3	7	8.5	9.25	3.61E-02
Aflatoxin B1 aldehyde reductase member 3	ARK73_RAT	P38918	0	0.5	1.5	9	4.5	4	8.75	3.51E-02
Coatomer subunit beta'	COPB2_RAT	O35142	0.5	0.5	1.5	4	8.5	8.5	8.40	1.60E-02
14-3-3 protein epsilon	1433E_RAT	P62260	0	1.5	0.5	7	2.5	4.5	7.00	4.37E-02
Kynurenine/alpha-aminoadipate aminotransferase, mitochondrial	AADAT_RAT	Q64602	0	1	1	3.5	4	6	6.75	1.00E-02
Thioredoxin	THIO_RAT	P11232	4.5	2.5	4.5	23	19	32.5	6.48	6.63E-03
C4b-binding protein alpha chain	C4BPA_RAT	Q63514	1	0	1	2	4.5	6	6.25	4.48E-02
Cluster of Glutathione S-transferase omega-1	GSTO1_RAT [2]	Q9Z339	0.5	1	1.5	5	6	7.5	6.17	2.72E-03
Class I histocompatibility antigen, Non-RT1.A alpha-1 chain	HA11_RAT	P15978	5.5	6	5	26	35	38	6.00	1.61E-03
Transforming protein RhoA	RHOA_RAT	P61589	0	2	0	5.5	3	3.5	6.00	3.03E-02
Neutrophil collagenase	MMP8_RAT	O88766	0	3	0	3.5	7	6.5	5.67	3.45E-02
Protein disulfide-isomerase A4	PDIA4_RAT	P38659	1	1	1	3.5	4	6.5	4.67	1.68E-02
Superoxide dismutase [Cu-Zn]	SODC_RAT	P07632	23	10	9	57.5	59	77.5	4.62	2.97E-03
RT1 class I histocompatibility antigen, AA alpha chain	HA12_RAT	P16391	5.5	8	4	23	23.5	32	4.49	2.95E-03
Selenium-binding protein 1	SBP1_RAT	Q8VIF7	0	2	2.5	5	7.5	7.5	4.44	1.03E-02

Tumor necrosis factor receptor superfamily member 1B	TNR1B_RAT	Q80WY6	1	1.5	1.5	6	6	5.5	4.38	4.43E-05
1,2-dihydroxy-3-keto-5-methylthiopentene dioxygenase	MTND_RAT	Q562C9	3	3.5	3.5	14	11	16.5	4.15	2.78E-03
Alpha/beta hydrolase domain-containing protein 14B	ABHEB_RAT	Q6DGG1	4	7.5	6	23	26	22.5	4.09	2.70E-04
Elongation factor 1-alpha 1	EF1A1_RAT	P62630	1	2	3.5	9.5	6.5	10.5	4.08	8.99E-03
Vascular cell adhesion protein 1	VCAM1_RAT	P29534	3.5	2.5	1.5	8	12.5	10	4.07	5.75E-03
Moesin	MOES_RAT	O35763	7.5	5	6	31	14	24	3.73	2.79E-02
Complement C4	CO4_RAT	P08649	4.5	7.5	14.5	32	25.5	37.5	3.58	7.46E-03
Pancreatic secretory granule membrane major glycoprotein GP2	GP2_RAT	P19218	0.5	5.5	0.5	7	7.5	8.5	3.54	3.32E-02
Ezrin	EZRI_RAT	P31977	7	8	5.5	25	14.5	30	3.39	2.42E-02
Phospholysine phosphohistidine inorganic pyrophosphate phosphatase	LHPP_RAT	Q5I0D5	4	5.5	2.5	12.5	14	13	3.29	7.04E-04
D-dopachrome decarboxylase	DOPD_RAT	P80254	2	3	2.5	9.5	6.5	8.5	3.27	3.64E-03
Na(+)/H(+) exchange regulatory cofactor NHE-RF1	NHRF1_RAT	Q9JJ19	8	4.5	10.5	31	15	25.5	3.11	3.20E-02
Protein deglycase DJ-1	PARK7_RAT	O88767	5.5	3.5	5	11	8.5	15	2.46	2.63E-02
Collectin-12	COL12_RAT	Q4V885	3.5	0.5	1.5	4.5	4.5	4.5	2.45	3.90E-02
Na(+)/H(+) exchange regulatory cofactor NHE-RF3	NHRF3_RAT	Q9JJ40	16	8.5	15.5	38.5	21	33.5	2.33	3.70E-02
Cluster of Actin, cytoplasmic 1	ACTB_RAT [4]	P60711	11	18	22	35	36.5	42.5	2.24	6.01E-03
Cluster of 14-3-3 protein zeta/delta	1433Z_RAT [5]	P63102	5	5	7.5	16.5	9	12.5	2.17	4.22E-02

Galectin-3-binding protein	LG3BP_RAT	O70513	14	11.5	13	25	30	21	1.97	9.85E-03
Alpha-1-acid glycoprotein	A1AG_RAT	P02764	14	13	22	34	29.5	32	1.95	7.76E-03
Galectin-5	LEG5_RAT	P47967	10	8	8.5	14	15	22.5	1.94	3.87E-02
Intercellular adhesion molecule 1	ICAM1_RAT	Q00238	6.5	4.5	5.5	9	13.5	9	1.91	3.58E-02
Neprilysin	NEP_RAT	P07861	13	12.5	16	31.5	18.5	28	1.88	3.93E-02
Uromodulin	UROM_RAT	P27590	83	56.5	81	113.5	135	138.5	1.76	8.65E-03
Complement factor D	CFAD_RAT	P32038	26.5	20	24.5	16.5	11.5	18	0.65	3.87E-02
Calbindin	CALB1_RAT	P07171	11	10.5	12.5	6	7.5	8.5	0.65	1.32E-02
Clusterin	CLUS_RAT	P05371	16	18.5	17.5	13	8.5	11.5	0.63	1.37E-02
Serum albumin	ALBU_RAT	P02770	257	278.5	308	194.5	158.5	173	0.62	4.27E-03
CD320 antigen	CD320_RAT	Q5HZW5	5	4.5	3.5	2	3	3	0.62	3.94E-02
Matrix-remodeling-associated protein 8	MXRA8_RAT	Q5XI43	29	29.5	20.5	13.5	20	13	0.59	4.25E-02
Lysosomal acid phosphatase	PPAL_RAT	P20611	14.5	12	16	8.5	9.5	5.5	0.55	1.94E-02
Gamma-interferon-inducible lysosomal thiol reductase	GILT_RAT	Q499T2	18	13.5	18.5	9.5	9.5	8	0.54	1.00E-02
Osteopontin	OSTP_RAT	P08721	9.5	8	9.5	3	4	7.5	0.54	4.56E-02
Lysosomal alpha-glucosidase	LYAG_RAT	Q6P7A9	14	21	18	8	11.5	8	0.52	2.21E-02
Cluster of Carboxylesterase 1C	EST1C_RAT [4]	P10959	14	14	16.5	4.5	9.5	8	0.49	1.16E-02
Serine protease inhibitor A3L	SPA3L_RAT	P05544	159.5	145.5	116	45.5	92.5	51.5	0.45	1.69E-02
Glutathione peroxidase 3	GPX3_RAT	P23764	6.5	6	5.5	3	3.5	1.5	0.44	7.49E-03
Beta-2-glycoprotein 1	APOH_RAT	P26644	14.5	10	13	4.5	7.5	4.5	0.44	1.35E-02
Probasin	PBAS_RAT	P15399	14.5	14	17.5	2.5	6	11.5	0.43	3.79E-02
Nucleobindin-1	NUCB1_RAT	Q63083	17	18	14	9	6	4	0.39	6.07E-03
Apolipoprotein E	APOE_RAT	P02650	18	17.5	20.5	9	4	8	0.38	2.85E-03

Attractin	ATRN_RAT	Q99J86	10.5	14	13	6	6.5	1.5	0.37	1.46E-02
Fetuin-B	FETUB_RAT	Q9QX79	20	21.5	18	7	6.5	8.5	0.37	4.47E-04
Vitamin D-binding protein	VTDB_RAT	P04276	42	42.5	51	19.5	19.5	11	0.37	2.19E-03
Lysosomal thioesterase PPT2	PPT2_RAT	O70489	5.5	6.5	5	0.5	4	1.5	0.35	3.16E-02
Growth arrest-specific protein 6	GAS6_RAT	Q63772	6.5	7.5	6	1	4.5	1.5	0.35	2.13E-02
Dipeptidyl peptidase 1	CATC_RAT	P80067	12.5	15	8.5	3	6.5	2	0.32	2.49E-02
Transthyretin	TTHY_RAT	P02767	9.5	6.5	8.5	2	3.5	2	0.31	5.03E-03
C-reactive protein	CRP_RAT	P48199	6.5	9.5	6	0.5	3.5	2.5	0.30	2.12E-02
Retinol-binding protein 4	RET4_RAT	P04916	5.5	3.5	5	1.5	1	1.5	0.29	5.90E-03
Putative phospholipase B-like 2	PLBL2_RAT	Q4QQW8	6	9	4.5	1.5	3.5	0.5	0.28	4.26E-02
Beta-microseminoprotein	MSMB_RAT	P97580	7.5	10	8.5	0.5	4	2.5	0.27	7.09E-03
Urinary protein 2	UP2_RAT	P81828	132	104.5	105	33.5	20	26.5	0.23	9.13E-04
Aggrecan core protein	PGCA_RAT	P07897	14.5	14	10.5	3.5	2.5	3	0.23	1.50E-03
Cluster of Alpha-1-inhibitor 3	A1I3_RAT [4]	P14046	54.5	84.5	100.5	13.5	31.5	8.5	0.22	1.51E-02
Follistatin-related protein 1	FSTL1_RAT	Q62632	8	8	8.5	0.5	4	0.5	0.20	5.27E-03
Complement factor I	CFAI_RAT	Q9WUW3	4	4.5	7	3	0	0	0.19	3.79E-02
Urinary protein 3	UP3_RAT	P83121	98.5	89	89	19.5	5.5	17.5	0.15	1.33E-04
Beta-defensin 50	DFB50_RAT	Q30KJ2	5	4	4.5	0	1	0.5	0.11	6.08E-04
Activin receptor type-1B	ACV1B_RAT	P80202	6	4	3	0	0	0	0.00	7.97E-03
Apolipoprotein A-IV	APOA4_RAT	P02651	6	8.5	9	0	0	0	0.00	1.08E-03

Day 9

Protein name	Accession Number	Uniprot ID	C-1-ave	C-2-ave	C-3-ave	D9-1-ave	D9-2-ave	D9-3-ave	Fold change	P-value
Ig gamma-2B chain C region	IGG2B_RAT	P20761	0	1.5	1	15	18	12	18.00	1.37E-03
Acidic mammalian chitinase	CHIA_RAT	Q6RY07	0.5	1	0	7	7	9.5	15.67	1.14E-03
Neutrophil gelatinase-associated lipocalin	NGAL_RAT	P30152	1.5	4	4	35	45	32.5	11.84	9.26E-04
Cluster of Keratin, type II cytoskeletal 8	K2C8_RAT [2]	Q10758	0	1.5	0	5	4	8.5	11.67	2.14E-02
NKG2-D type II integral membrane protein	NKG2D_RAT	O70215	0	1.5	0	4.5	4	5.5	9.33	3.34E-03
Class I histocompatibility antigen, Non-RT1.A alpha-1 chain	HA11_RAT	P15978	5.5	6	5	30	36	52.5	7.18	7.23E-03
Transforming protein RhoA	RHOA_RAT	P61589	0	2	0	4	3	5	6.00	1.94E-02
RT1 class I histocompatibility antigen, AA alpha chain	HA12_RAT	P16391	5.5	8	4	23.5	28	38	5.11	5.68E-03
Na(+)/H(+) exchange regulatory cofactor NHE-RF1	NHRF1_RAT	Q9JJ19	8	4.5	10.5	35.5	41.5	36	4.91	3.19E-04
Brain acid soluble protein 1	BASP1_RAT	Q05175	2.5	0.5	1.5	6	7.5	6.5	4.44	2.07E-03
Tumor necrosis factor receptor superfamily member 1B	TNR1B_RAT	Q80WY6	1	1.5	1.5	6	6	5	4.25	3.13E-04
Moesin	MOES_RAT	O35763	7.5	5	6	20	29	29.5	4.24	3.23E-03
Ezrin	EZRI_RAT	P31977	7	8	5.5	20	27.5	33.5	3.95	7.10E-03

Alpha-1-acid glycoprotein	A1AG_RAT	P02764	14	13	22	48	66.5	68	3.72	3.19E-03
Chloride intracellular channel protein 1	CLIC1_RAT	Q6MG61	2	0	1.5	2.5	5.5	5	3.71	4.57E-02
Vascular cell adhesion protein 1	VCAM1_RAT	P29534	3.5	2.5	1.5	6.5	8	10.5	3.33	1.10E-02
Cluster of Ig gamma-2A chain C region	IGG2A_RAT [2]	P20760	13	15	8.5	44	43.5	34	3.33	1.69E-03
Peptidyl-prolyl cis-trans isomerase A	PPIA_RAT	P10111	2.5	3.5	2.5	6	8	12	3.06	3.14E-02
Na(+)/H(+) exchange regulatory cofactor NHE-RF3	NHRF3_RAT	Q9JJ40	16	8.5	15.5	29.5	41.5	39.5	2.76	6.08E-03
Cluster of Putative lysozyme C-2	LYSC2_RAT [2]	Q05820	2.5	1	3	8	5	4.5	2.69	4.24E-02
Beta-2-microglobulin	B2MG_RAT	P07151	13.5	6.5	6	19.5	24	24	2.60	8.30E-03
Ig kappa chain C region, B allele	KACB_RAT	P01835	100.5	156.5	86.5	356	247	285	2.59	9.16E-03
Superoxide dismutase [Cu-Zn]	SODC_RAT	P07632	23	10	9	25	40	38.5	2.46	3.54E-02
Protein RoBo-1	ROB1_RAT	O55006	5.5	4.5	4.5	11.5	11	11	2.31	7.03E-05
Ribonuclease 4	RNAS4_RAT	O55004	7	3.5	4	13.5	8.5	11	2.28	2.71E-02
Cathepsin S	CATS_RAT	Q02765	4	3.5	2.5	7	7	8.5	2.25	3.34E-03
Cluster of Keratin, type II cytoskeletal 1	K2C1_RAT [3]	Q6IMF3	2.5	8	5	13	10	10.5	2.16	3.11E-02
Ig kappa chain C region, A allele	KACA_RAT	P01836	112.5	100.5	74	183	202	226	2.13	3.05E-03
Gamma-glutamyltranspeptidase 1	GGT1_RAT	P07314	16.5	13	16.5	29.5	28.5	34.5	2.01	2.11E-03

Ribonuclease UK114	UK114_RAT	P52759	4	2.5	2	5.5	6.5	4.5	1.94	3.29E-02
Neprilysin	NEP_RAT	P07861	13	12.5	16	26.5	26	27.5	1.93	4.04E-04
Intercellular adhesion molecule 1	ICAM1_RAT	Q00238	6.5	4.5	5.5	7.5	13	10.5	1.88	4.60E-02
Cluster of 14-3-3 protein zeta/delta	1433Z_RAT [5]	P63102	5	5	7.5	10	10.5	12	1.86	8.24E-03
Galectin-3-binding protein	LG3BP_RAT	O70513	14	11.5	13	24	27.5	20	1.86	8.56E-03
Ig lambda-2 chain C region	LAC2_RAT	P20767	26	28	31	52.5	57	45.5	1.82	3.07E-03
Polymeric immunoglobulin receptor	PIGR_RAT	P15083	28.5	31.5	32	46	55.5	63	1.79	8.67E-03
Protein deglycase DJ-1	PARK7_RAT	O88767	5.5	3.5	5	9.5	8.5	7	1.79	1.77E-02
Cluster of Heat shock cognate 71 kDa protein	HSP7C_RAT [4]	P63018	14	11.5	11.5	24.5	16.5	21.5	1.69	2.65E-02
Granulins	GRN_RAT	P23785	13.5	13.5	16	20.5	22	27	1.62	1.44E-02
Sialate O-acetylesterase	SIAE_RAT	P82450	7	7.5	4	9.5	9	9.5	1.51	4.57E-02
Serum albumin	ALBU_RAT	P02770	257	278.5	308	183	216	141	0.64	1.83E-02
Matrix-remodeling-associated protein 8	MXRA8_RAT	Q5XI43	29	29.5	20.5	16.5	18.5	14.5	0.63	3.51E-02
Attractin	ATRN_RAT	Q99J86	10.5	14	13	9.5	7.5	6	0.61	2.92E-02
Neogenin (Fragment)	NEO1_RAT	P97603	4.5	5.5	5	3	3	3	0.60	2.28E-03
Pro-epidermal growth factor	EGF_RAT	P07522	128	149.5	144.5	75.5	78.5	94	0.59	2.59E-03
Protein FAM151A	F151A_RAT	Q642A7	6.5	7.5	6.5	2.5	5	4.5	0.59	2.73E-02
Transthyretin	TTHY_RAT	P02767	9.5	6.5	8.5	3	5.5	5.5	0.57	4.48E-02
Apolipoprotein E	APOE_RAT	P02650	18	17.5	20.5	8.5	12	10	0.54	3.47E-03
Alpha-1-macroglobulin	A1M_RAT	Q63041	97	124.5	130.5	76.5	60	49.5	0.53	1.30E-02

Deoxyribonuclease-1	DNAS1_RAT	P21704	28	41	28.5	21.5	14.5	15.5	0.53	3.27E-02
Urinary protein 2	UP2_RAT	P81828	132	104.5	105	48	60.5	68.5	0.52	7.25E-03
Nidogen-1 (Fragment)	NID1_RAT	P08460	11.5	12	10.5	4.5	7	6	0.51	2.94E-03
Dipeptidyl peptidase 2	DPP2_RAT	Q9EPB1	11.5	13	17.5	8	6	7	0.50	2.09E-02
Lysosomal alpha-glucosidase	LYAG_RAT	Q6P7A9	14	21	18	9	9	7.5	0.48	1.18E-02
Serine protease inhibitor A3L	SPA3L_RAT	P05544	159.5	145.5	116	41	63.5	85.5	0.45	1.32E-02
Growth arrest-specific protein 6	GAS6_RAT	Q63772	6.5	7.5	6	2.5	3	3.5	0.45	2.24E-03
Nucleobindin-1	NUCB1_RAT	Q63083	17	18	14	2.5	11	8.5	0.45	3.22E-02
Urinary protein 3	UP3_RAT	P83121	98.5	89	89	33	37.5	51.5	0.44	1.30E-03
Vitamin D-binding protein	VTDB_RAT	P04276	42	42.5	51	18.5	28.5	11	0.43	1.15E-02
Aggrecan core protein	PGCA_RAT	P07897	14.5	14	10.5	3	6	7.5	0.42	1.48E-02
Beta-2-glycoprotein 1	APOH_RAT	P26644	14.5	10	13	4.5	8.5	2.5	0.41	2.92E-02
Fetuin-B	FETUB_RAT	Q9QX79	20	21.5	18	5.5	9	10	0.41	2.36E-03
Vomeromodulin (Fragment)	VOME_RAT	Q63751	10.5	10.5	7	3.5	5	2.5	0.39	1.46E-02
Cubilin	CUBN_RAT	O70244	27.5	45.5	29.5	20	9.5	10	0.39	3.42E-02
Procollagen C-endopeptidase enhancer 1	PCOC1_RAT	O08628	3	5	5	2.5	2	0.5	0.38	4.11E-02
Anthrax toxin receptor 1	ANTR1_RAT	Q0PMD2	5	5	4.5	1.5	2.5	1.5	0.38	1.29E-03
Sulfhydryl oxidase 1	QSOX1_RAT	Q6IUU3	13.5	19.5	14.5	7	6.5	4.5	0.38	8.05E-03
Biotinidase	BTD_RAT	Q5FVF9	12.5	13.5	12.5	5.5	4	5	0.38	1.33E-04
Low-density lipoprotein receptor-related protein 2	LRP2_RAT	P98158	118	158.5	158	94.5	43	25.5	0.38	2.14E-02
Calbindin	CALB1_RAT	P07171	11	10.5	12.5	2.5	7.5	2	0.35	1.68E-02
Retinol-binding protein 4	RET4_RAT	P04916	5.5	3.5	5	1.5	1	2	0.32	8.97E-03
C-reactive protein	CRP_RAT	P48199	6.5	9.5	6	1.5	3	2	0.30	1.18E-02

Dipeptidyl peptidase 1	CATC_RAT	P80067	12.5	15	8.5	4.5	4	2	0.29	1.41E-02
Cluster of Carboxylesterase 1C	EST1C_RAT [4]	P10959	14	14	16.5	4	5	3	0.27	4.34E-04
Follistatin-related protein 1	FSTL1_RAT	Q62632	8	8	8.5	1	3	2	0.24	5.08E-04
Multiple inositol polyphosphate phosphatase 1	MINP1_RAT	O35217	10.5	12.5	11.5	4	1.5	2.5	0.23	6.80E-04
Serine protease inhibitor A3M (Fragment)	SPA3M_RAT	Q63556	17	7	13.5	2	4.5	1.5	0.21	3.29E-02
Beta-defensin 50	DFB50_RAT	Q30KJ2	5	4	4.5	0	1	1.5	0.19	2.24E-03
Cluster of Alpha-1-inhibitor 3	A1I3_RAT [4]	P14046	54.5	84.5	100.5	16	16.5	3	0.15	8.69E-03
Sialidase-1	NEUR1_RAT	Q99PW3	7	8	6.5	0.5	1.5	1	0.14	3.05E-04
Apolipoprotein A-IV	APOA4_RAT	P02651	6	8.5	9	0	2	1	0.13	3.34E-03

Supplementary Table S6. Differential proteins identified in different stages in 7 Walker-256 tail-vein injection rats.

Time points	Common identified	Triple TOF 5600™	Orbitrap Fusion Lumos
**D2**	APOE_RAT,LG3BP_RAT,EGF_RAT,NID2_RAT,A1M_RAT,ABHEB_RAT,UP3_RAT	MTND_RAT,IC1_RAT,PIGR_RAT,AMPN_RAT,PRDX6_RAT,HSP7C_RAT,CK054_RAT,MXRA8_RAT,IF6_RAT,CO4_RAT,ANTR1_RAT,GSTO1_RAT,UP2_RAT,EZRI_RAT,ACY1A_RAT,DPP4_RAT,LEG5_RAT,KLK1_RAT,PIP_RAT	ACV1B_RAT,NHRF1_RAT,NEO1_RAT,NID1_RAT,GELS_RAT,CATC_RAT,PCOC1_RAT,FETUB_RAT,EST1C_RAT[4],ANGT_RAT,ALBU_RAT,ATRN_RAT,GAS6_RAT,VTDB_RAT,APOA4_RAT,PPT2_RAT,NGAL_RAT,THIO_RAT,HA12_RAT,KACA_RAT,UROM_RAT,RET4_RAT,GP2_RAT,HA11_RAT,BTD_RAT,NEUR1_RAT,ENOA_RAT,LEG9_RAT,PPIA_RAT,SPA3L_RAT,RHOA_RAT,MINP1_RAT,NTF2_RAT,BASP1_RAT,GGT1_RAT,PDIA1_RAT
**D4**	FETUB_RAT,ALBU_RAT,NGAL_RAT,BTD_RAT,A1AG_RAT,PIGR_RAT,CATC_RAT,LG3BP_RAT,VTDB_RAT,HA12_RAT,CO4_RAT,A1M_RAT,SPA3L_RAT,GGT1_RAT,UP3_RAT,ROB1_RAT	SLC31_RAT,AMPN_RAT,HSP7C_RAT,MOES_RAT,NHRF3_RAT,MXRA8_RAT,EGF_RAT,ALDOB_RAT,TRFE_RAT,ABHEB_RAT,ICAM1_RAT,SPA3K_RAT,GSTO1_RAT,ACY1A_RAT,KLK1_RAT,LEG5_RAT,PIP_RAT,MTND_RAT,CALB1_RAT,IC1_RAT,CLIC1_RAT,GSH1_RAT,EF1A1_RAT,KLK7_RAT,CD14_RAT,PRVA_RAT,UROM_RAT,VDAC1_RAT,FETUA_RAT,AMPE_RAT,F16P1_RAT,CFAD_RAT,EST1C_RAT,EZRI_RAT,CRP_RAT,DPP4_RAT,GGH_RAT,ACTG_RAT	NKG2D_RAT,GDIR1_RAT,EST1C_RAT[4],TTHY_RAT,DFB50_RAT,GAS6_RAT,APOA4_RAT,THIO_RAT,RET4_RAT,ACY3_RAT,UP2_RAT,APOH_RAT,A1I3_RAT[4],ATRN_RAT,PPT2_RAT,GPC5C_RAT,HA11_RAT,NEP_RAT

**D6**	MOES_RAT,MXRA8_RAT,FETUB_RAT,ABHEB_RAT,ICAM1_RAT,A1AG_RAT,LEG5_RAT,MTND_RAT,CALB1_RAT,APOE_RAT,CATC_RAT,LG3BP_RAT,VTDB_RAT,HA12_RAT,UROM_RAT,HA11_RAT,CO4_RAT,SODC_RAT,CFAD_RAT,SPA3L_RAT,EZRI_RAT,UP3_RAT	THRB_RAT,HSP7C_RAT,CDHR5_RAT,EGF_RAT,BTD_RAT,6PGL_RAT,SPA3K_RAT,ACY1A_RAT,KLK1_RAT,PIP_RAT,IC1_RAT,CLIC1_RAT,PIGR_RAT,MUG1_RAT,CD14_RAT,A1I3_RAT,IF6_RAT,K2C1_RAT,VDAC1_RAT,F16P1_RAT,A1M_RAT,MEP1A_RAT,EST1C_RAT,DPP4_RAT,GGH_RAT,ACTG_RAT,ROB1_RAT	ACV1B_RAT,LHPP_RAT,1433Z_RAT[5],CAH1_RAT,CFAI_RAT,NKG2D_RAT,NUCB1_RAT,THTM_RAT,PLBL2_RAT,NHRF3_RAT,SBP1_RAT,CLIC4_RAT,GPX3_RAT,EST1C_RAT[4],FABP7_RAT,TTHY_RAT,ALBU_RAT,DFB50_RAT,GAS6_RAT,PPAL_RAT,APOA4_RAT,NGAL_RAT,THIO_RAT,RET4_RAT,GP2_RAT,PGCA_RAT,CD320_RAT,PTGR2_RAT,TNR1B_RAT,MMP8_RAT,LYPA1_RAT,CLUS_RAT,VCAM1_RAT,RET1_RAT,ATPB_RAT,UP2_RAT,ACTB_RAT[4],GSTO1_RAT[2],APOH_RAT,AADAT_RAT,NHRF1_RAT,COL12_RAT,GILT_RAT,FSTL1_RAT,ENPP3_RAT,OSTP_RAT,EF1A1_RAT,DOPD_RAT,CIAO1_RAT,MSMB_RAT,SCRN2_RAT,CYTB_RAT,A1I3_RAT[4],ATRN_RAT,COPB2_RAT,PARK7_RAT,PPT2_RAT,LYAG_RAT,C4BPA_RAT,1433E_RAT,ARK73_RAT,CA123_RAT,RHOA_RAT,CRP_RAT,NEP_RAT,IDI1_RAT,PBAS_RAT,PDIA4_RAT

**D9**	CATS_RAT,MOES_RAT,NHRF3_RAT,MXRA8_RAT,FETUB_RAT,EGF_RAT,ALBU_RAT,APOA4_RAT,NGAL_RAT,SIAE_RAT,BTD_RAT,ICAM1_RAT,A1AG_RAT,NHRF1_RAT,CALB1_RAT,LAC2_RAT,IGG2B_RAT,CLIC1_RAT,APOE_RAT,PIGR_RAT,LG3BP_RAT,LRP2_RAT,PARK7_RAT,HA12_RAT,LYAG_RAT,SODC_RAT,A1M_RAT,SPA3L_RAT,EZRI_RAT,CRP_RAT,GGT1_RAT,UP3_RAT,ROB1_RAT,KACB_RAT	SLC31_RAT,THRB_RAT,CATL1_RAT,AMPN_RAT,PRDX6_RAT,HSP7C_RAT,PEBP1_RAT,CK054_RAT,CDHR5_RAT,PLBL2_RAT,FABP7_RAT,IGG2A_RAT,CD48_RAT,CTL4_RAT,ABHEB_RAT,SPA3K_RAT,ACY1A_RAT,CBG_RAT,KLK1_RAT,PIP_RAT,MTND_RAT,IC1_RAT,GSH1_RAT,MUG1_RAT,KVX01_RAT,CAH3_RAT,KLK7_RAT,FINC_RAT,CD14_RAT,A1I3_RAT,PRVA_RAT,IF6_RAT,K2C1_RAT,VDAC1_RAT,FETUA_RAT,F16P1_RAT,CO4_RAT,MEP1A_RAT,EST1C_RAT,IDI1_RAT,NTF2_RAT,DPP4_RAT,GGH_RAT,ACTG_RAT	NID1_RAT,NEO1_RAT,HSP7C_RAT[4],1433Z_RAT[5],QSOX1_RAT,B2MG_RAT,DPP2_RAT,NKG2D_RAT,F151A_RAT,NUCB1_RAT,K2C8_RAT[2],VOME_RAT,EST1C_RAT[4],TTHY_RAT,DFB50_RAT,GAS6_RAT,RET4_RAT,PGCA_RAT,TNR1B_RAT,NEUR1_RAT,VCAM1_RAT,PPIA_RAT,ANTR1_RAT,MINP1_RAT,UP2_RAT,APOH_RAT,GRN_RAT,FSTL1_RAT,DNAS1_RAT,CATC_RAT,A1I3_RAT[4],CHIA_RAT,K2C1_RAT[3],PCOC1_RAT,RNAS4_RAT,CUBN_RAT,SPA3M_RAT,ATRN_RAT,VTDB_RAT,LYSC2_RAT[2],KACA_RAT,HA11_RAT,UK114_RAT,IGG2A_RAT[2],RHOA_RAT,NEP_RAT,BASP1_RAT

Supplementary Table S7. Twenty differential proteins enriched in biological process associated with immune system.

Biological process	Uniprot ID	Gene
	IGG2B_RAT	Igh-1a
	IC1_RAT	Serping1
	CFAI_RAT	Cfi
	CO4_RAT	C4
	IGG2A_RAT	Igg-2a
**regulation of immune system process**	SIAE_RAT	Siae
**complement activation, classical pathway**	A1AG_RAT	Orm1
**ERK1 and ERK2 cascade**	GGT1_RAT	Ggt1
**innate immune response**	PIP_RAT	Pip
**positive regulation of B cell activation**	CRP_RAT	Crp
**B cell receptor signaling pathway**	CO9_RAT	C9
	APOA1_RAT	Apoa1
	TRFE_RAT	Tf
	LEG9_RAT	Lgals9
	ANGT_RAT	Agt
	EGF_RAT	Egf
	NGAL_RAT	Lcn2
	B2MG_RAT	B2m
	NKG2D_RAT	Klrk1
	CD14_RAT	Cd14
